# Generating Executable Models of the Drosophila Central Complex

**DOI:** 10.3389/fnbeh.2017.00102

**Published:** 2017-05-30

**Authors:** Lev E. Givon, Aurel A. Lazar, Chung-Heng Yeh

**Affiliations:** ^1^The Charles Stark Draper Laboratory, Inc.Cambridge, MA, United States; ^2^Bionet Group, Department of Electrical Engineering, Columbia UniversityNew York, NY, United States

**Keywords:** central complex, Drosophila, brain emulation, visualization

## Abstract

The central complex (CX) is a set of neuropils in the center of the fly brain that have been implicated as playing an important role in vision-mediated behavior and integration of spatial information with locomotor control. In contrast to currently available data regarding the neural circuitry of neuropils in the fly's vision and olfactory systems, comparable data for the CX neuropils is relatively incomplete; many categories of neurons remain only partly characterized, and the synaptic connectivity between CX neurons has yet to be fully determined. Successful modeling of the information processing functions of the CX neuropils therefore requires a means of easily constructing and testing a range of hypotheses regarding both the high-level structure of their neural circuitry and the properties of their constituent neurons and synapses. To this end, we have created a web application that enables simultaneous graphical querying and construction of executable models of the CX neural circuitry based upon currently available information regarding the geometry and polarity of the arborizations of identified local and projection neurons in the CX. The application's novel functionality is made possible by the Fruit Fly Brain Observatory, a platform for collaborative study and development of fruit fly brain models.

## 1. Introduction

**Figure 1:**
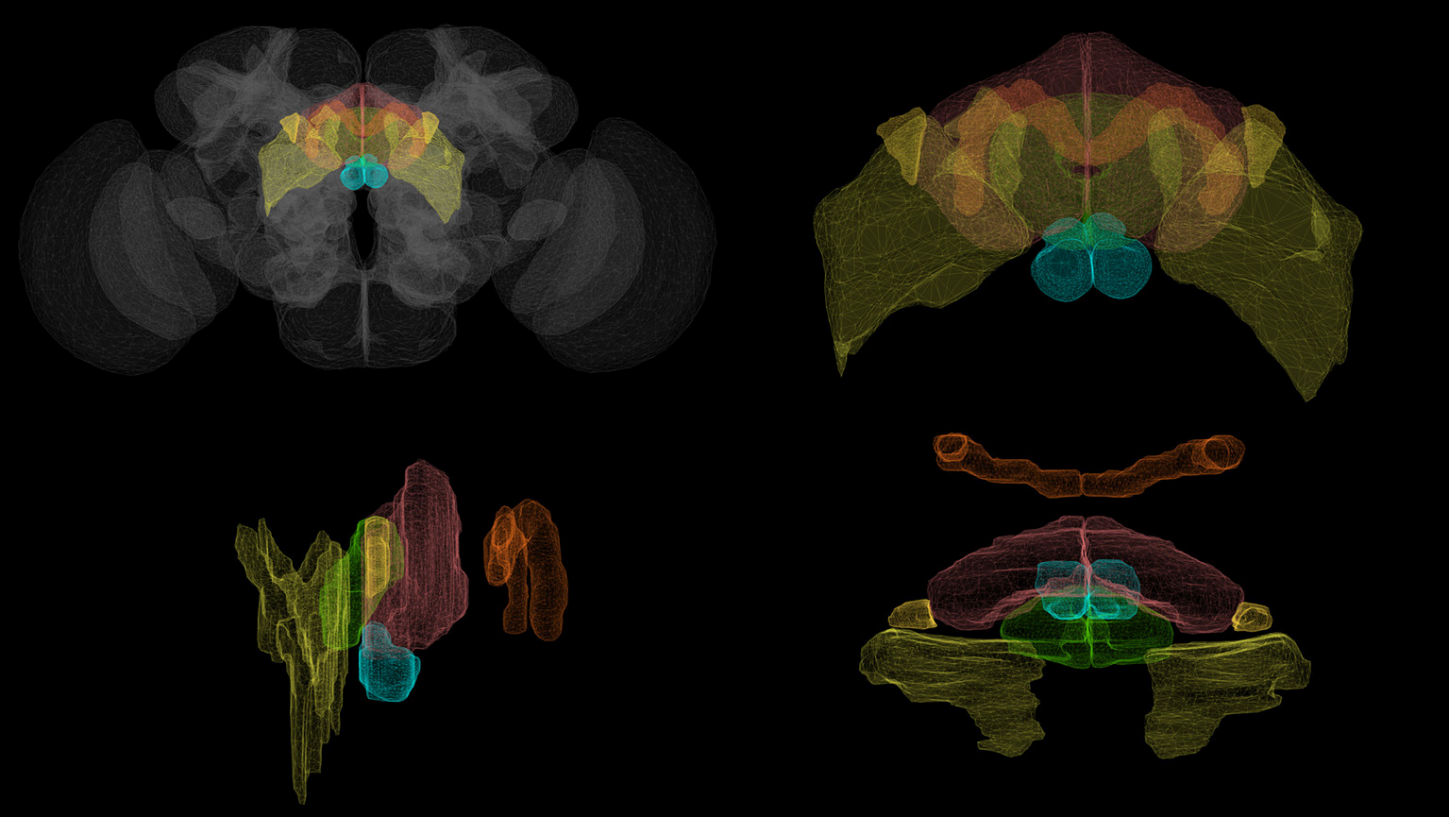
Volumetric rendering of central complex neuropils (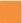
PB, 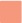
FB, 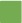
EB, 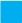
NO) and select accessory neuropils (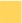
BU, 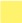
LAL) innervated by CX neurons. (Clockwise from top left: whole brain, front view of the central complex, side view of the central complex, top view of the central complex.) Rendering created with NeuroGFX using volumetric information from the FlyCircuit database (Chiang et al., 2011).

The brain of the fruit fly *Drosophila melanogaster* comprises approximately 50 neuropils. Most of these modules—referred to as local processing units (LPUs) are characterized by unique populations of local neurons; some—called hubs—do not contain any local neurons (Chiang et al., 2011). The central complex (CX) comprises between 2,000 and 5,000 neurons (Strauss, 2014) organized in four neuropils: the protocerebral bridge (PB), fan-shaped body (FB), ellipsoid body (EB), and noduli (NO) (Figure 1). Local neurons have been identified in PB and FB, but not in EB or NO (Chiang et al., 2011; Wolff et al., 2015). In contrast to most neuropils in the fly brain, PB, FB, and EB are unpaired; NO comprises 3 paired subunits (Wolff et al., 2015). Accessory brain areas that are connected directly to neuropils in CX include the bulb (BU), crepine (CRE), inferior bridge (IB), lateral accessory lobe (LAL), superior medial protocerebrum (SMP), wedge (WED), and posterior slope (PS) (Lin et al., 2013).

Although, a growing amount of CX structural information is available for several insect species other than the fruit fly such as the monarch butterfly, desert locust, field cricket, and discoid cockroach (Pfeiffer and Homberg, 2014), CX connectome information is currently less complete than that of sensory neuropils such as those in the olfactory and vision systems, the latter of which has recently been mapped in the fly in great detail using electron microscopy (Takemura, 2015). A range of local and projection neurons in CX have been identified and grouped into isomorphic sets using Golgi staining, genetic tagging techniques, and confocal microscopy (Hanesch et al., 1989; Young and Armstrong, 2010b; Lin et al., 2013; Wolff et al., 2015); however, many other CX neurons have not been systematically characterized and the synaptic connectivity between them remains unknown owing to the limitations of the above optical imaging technologies and the very limited EM-based analysis of CX synapses done to date (for an example of the latter, see Martín-Peña et al., 2014). This ambiguity regarding the structure of the CX neural circuitry compounds the already difficult task of modeling a portion of the brain that does not receive direct sensory input.

Genetic experiments have shown that the CX neuropils play essential roles in a range of important behaviors:

- EB appears to be involved in visual place learning (Ofstad et al., 2011; Dewar et al., 2015), short-term orientation memory (Neuser et al., 2008; Seelig and Jayaraman, 2013; Wystrach et al., 2014), angular path integration (Seelig and Jayaraman, 2015), and left-right bargaining (Strauss, 2014);
- FB appears to also play a role in left-right bargaining, as well as visual pattern memory and object recognition (Strauss and Berg, 2010; Strauss, 2014);
- PB plays a role in controlling step length and hence direction of walking (Strauss and Berg, 2010; Strauss, 2014);
- NO neuropils seem to be involved in flight control (Pfeiffer and Homberg, 2014).

While some functional models of the CX neuropils have been presented that attribute high-level functions such as short-term object storage and object recognition to different parts of the circuit (Strauss and Berg, 2010; Strauss, 2014), they do not explicitly show how the CX circuitry explicitly implements the information processing functions associated with the above behaviors or how the various neuropils' individual functions combine to produce more comprehensive behaviors such as long-term motor skill learning or locomotor activity control. In light of the incompleteness of the CX connectome, it is perhaps unsurprising that only a few computational models of the CX neuropils or the entire CX currently exist. A spiking neural network model of spatial memory formation and storage in EB is presented in Arena et al. (2013); while this model can replicate experimental results for specific behaviors using a ring attractor circuit inspired by that of EB, it does not attempt to account for the exact observed biological circuitry or explain how such a model interacts with the other CX neuropils. A model of CX was included in a more comprehensive insect brain simulation described in Arena et al. (2014), but it employs generalized models of the CX neuropils that use artificial behavior selection networks which—although they superficially make use of spiking neuron models— do not employ the observed neural circuitry of the neuropils.

To enable further investigation of the information processing capabilities of the CX neuropils, we need to be able to efficiently generate and evaluate different executable CX models given the limited available connectome data. While a similar approach involving *C. elegans* has been used to generate multiple testable models regarding the neural basis for salt klinotaxis behavior (Izquierdo and Beer, 2013), the greater structural complexity of the fruit fly CX and the need to evaluate the CX models together with models of the neuropils that provide them with input requires

- A database-driven approach to generating different models of the CX neural circuitry that incorporate experimentally obtained biological data with hypothetical or algorithmically inferred structural characteristics that attempt to account for the unknown aspects of the circuitry, and
- A graphical means of interacting with CX models and their outputs that exposes the circuitry at different levels of structural abstraction ranging from individual neurons through families of morphologically similar neurons to circuits comprising multiple neuron families.

To address these requirements, we have developed a web application for simultaneous graphical navigation of the CX and execution of models of its neural circuitry; this application may be accessed at http://fruitflybrain.org/neuroapps/central_complex. In this paper, we first describe the software architecture underlying this application and the unique visualization features of its user interface. We then present a scheme for labeling neurons in terms of their arborization patterns that can be used to algorithmically infer unknown synaptic connectivity in the CX neuropils. Finally, we demonstrate how this software utilizes this scheme to construct executable models comprising several families of neurons in the CX with two examples that, respectively, illustrate model responses to injected input signals and a comparison between the responses of models with circuitry based upon wild type and mutant fly strains.

## 2. Software architecture

**Figure 2:**
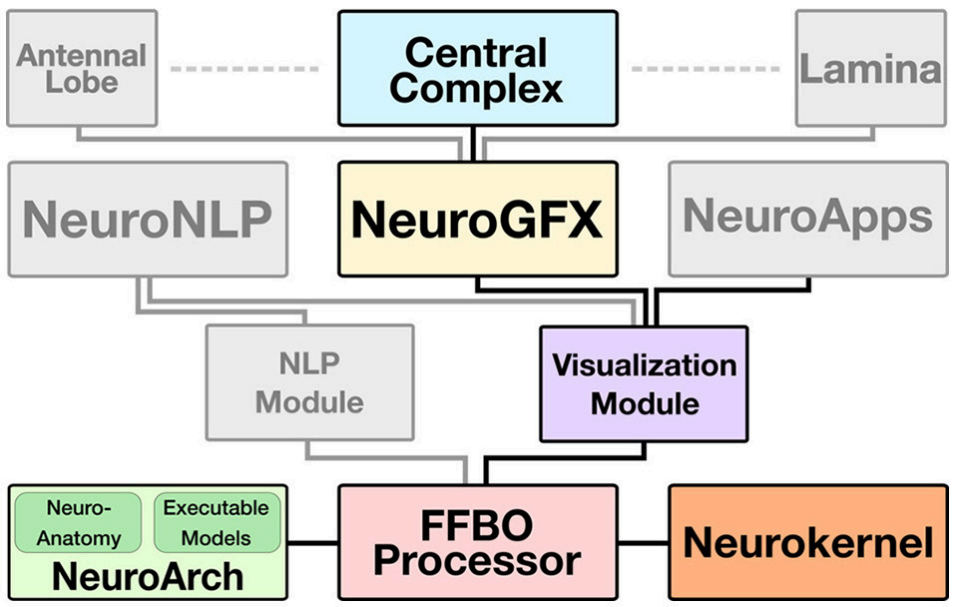
FFBO architecture for support of CX model visualization, manipulation, and execution. Solid lines depict data flow between software components. The **Central Complex Model** and those of other neuropils (e.g., **Antennal Lobe**, **Lamina**) are implemented as software applications that use **NeuroGFX** to support interactive user configuration and launching of model execution. The **Visualization Module** provides low-level routines for 3D rendering of neuropils and neuron morphologies utilized by NeuroGFX and comprehensive models of fly brian subsystems called **NeuroApps**. Neuroanatomy and executable circuit model data is stored in the **NeuroArch** database and efficiently executed by **Neurokernel**. The **FFBO Processor** sets up direct network connections between the other components of the architecture to accelerate data transfer during application execution. The **NLP (Natural Language Processing) Module** provides a high-level query interface to NeuroArch that is exposed to users through the **NeuroNLP** graphical interface.

**Figure 3:**
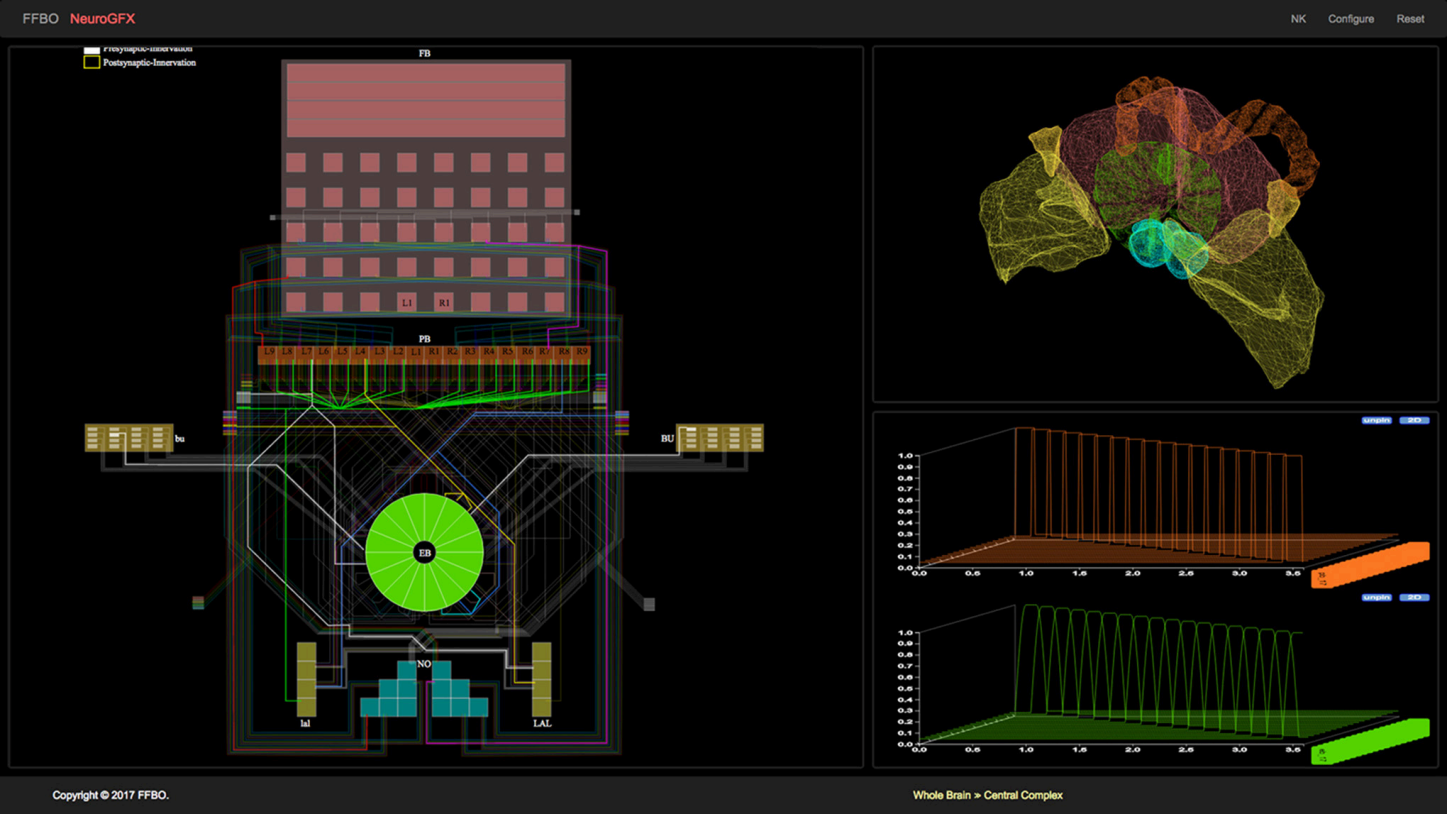
NeuroGFX graphical user interface depicting CX neural circuit, neuropils, and activity of neurons in executed CX model.

The CX web application is built upon several key software components that collectively constitute the **Fruit Fly Brain Observatory**, an open-source platform for the emulation and biological validation of fruit fly brain models in health and disease (Ukani et al., 2016b) (Figure 2):

- Fly brain circuit models are stored in **NeuroArch**, a graph database designed to facilitate the generation of executable neural circuit models (Givon et al., 2014). NeuroArch provides an extensible data model that unifies the representation of both biological and executable neural circuit data in a single graph. This data model currently supports a range of common point neuron and synapse models such as Leaky Integrate-and-Fire neurons, non-spiking Morris-Lecar neurons, and alpha function synapses. Circuits may be accessed via an object-graph mapping (OGM) interface that enables a range of sophisticated queries to be performed without having to explicitly specify complex query strings. This interface enables implementation of algorithms for inferring executable circuits from incomplete connectome data and sophisticated manipulation of stored neural circuit data to test model hypotheses. NeuroArch's query interface permits all circuit components' parameters to be modified regardless of whether they were algorithmically constructed.
- Fly brain models in NeuroArch may comprise local processing units that potentially contain different modeling components. To fuse these portions into a single executable model regardless of their internal design, the **Neurokernel** package defines a mandatory communication interface for neural circuit models exported by NeuroArch that enables their integration and execution on multiple graphics processing units (GPUs) models that utilize a range of neuron and synapse models defined by NeuroArch's current data model. Since Neurokernel's model communication interface is also described in NeuroArch's data model, circuits comprising multiple interconnected neuropils (such as the CX) may be fully specified in NeuroArch and immediately dispatched to Neurokernel for execution.
- **NeuroGFX** provides a reconfigurable graphical interface for navigation, manipulation, and execution of the CX neural circuit; a screenshot of this interface appears in Figure 3. (Yeh et al., 2016). Regions of neuropils comprised by and accessory to the central complex may be rendered in 3D. Neurons in the executed CX model may be selected and highlighted in a schematic circuit view; detailed portions of the circuit may also be magnified. The interface also enables multiple selected neuron responses to be concurrently plotted in 2D or 3D. Regions of the CX neuropils innervated by selected neurons may also be highlighted in real time as the model is executed. NeuroGFX's user interface is currently read-only; the capacity to modify stored CX models (via NeuroArch's query interface) will be added in the future.
- In addition to neuropil models exposed through NeuroGFX, the architecture supports development of customized graphical applications called **NeuroApps**. These provide access to specialized models of fly brain subsystems such as the vision or olfactory systems for exploring functions associated with models of healthy or diseased neural circuits in these systems.
- To provide both NeuroGFX and NeuroApps with the same base set of graphical features, the FFBO architecture provides a **Visualization Module** that contains routines for drawing neuropils and rendering neuron morphologies using WebGL.
- The **Natural Language Interface (NLP) Module** provides a user-friendly way to construct sophisticated queries against NeuroArch in plain English that obviate the need for users to directly interact with NeuroArch's OGM. This functionality is exposed to users through a graphical interface called **NeuroNLP** (Ukani et al., 2016a). NeuroNLP is not currently utilized by the CX model application.
- To accelerate data transmission between the above components and provide the modularity required to build future applications based upon the FFBO platform, the **FFBO Processor** sets up data connections between platform components that need to communication during application execution.

## 3. CX circuit representation and generation

### 3.1. Arborization-based neuron labeling

**Figure 4:**
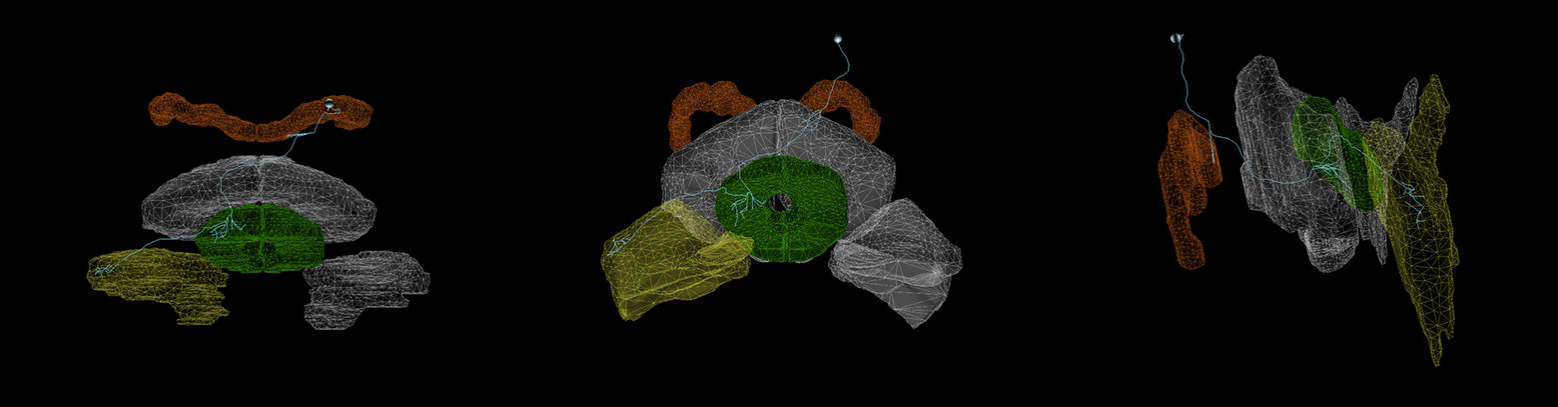
Arborizations of a PB-EB-LAL neuron (Wolff et al., 2015) (light blue) superimposed upon the CX neuropils (from left to right: top view, front view, side view.). Each arborization occupies a specific region of the PB, EB, and LAL neuropils (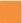
PB, 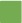
EB, 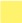
LAL). Rendering created with NeuroGFX using volumetric information of neuropils and skeletonic information of neurons from the FlyCircuit database (Chiang et al., 2011). This neuron is registered in the FlyCircuit database with the indentifier “Gad1-F-400245.”

Most neurons innervating the various CX and accessory neuropils possess at least two distinct clusters of dendrites (postsynaptic terminals) and/or axons (presynaptic terminals) that occupy geometrically distinct regions of the innervated neuropils (Hanesch et al., 1989). These clusters are referred to as arborizations (Figure 4). In the absence of experimental data regarding the actual presence and number of synapses between specific CX neurons, the overlap of presynaptic and postsynaptic arborizations may be used to infer synaptic connectivity and information flow until more detailed connectivity data becomes available. To use arborization data to infer synaptic connectivity, CX neurons with similar morphologies and arborization patterns can be classified and labeled in terms of the latter. If neurotransmitter profiles are ignored and each CX neuron type is assumed to be represented by a single neuron, then each neuron's label unambiguously encodes the geometric regions of its arborizations and whether each arborization contains dendrites, axons, or both.

**Figure 5:**
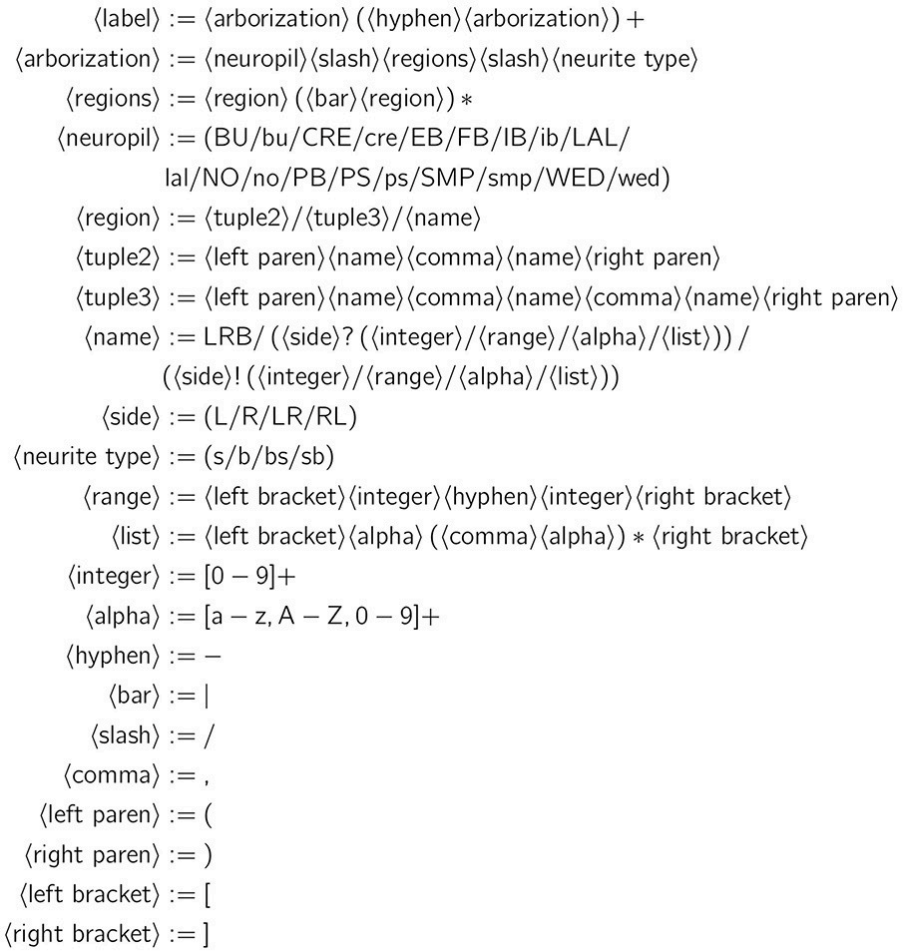
PEG grammar for CX neuron label.

This neuron labeling scheme can be described in terms of the parsing expression grammar (PEG) depicted in Figure 5 (Ford, 2004); the grammar may be used to extract the arborizations of a particular neuron for constructing models of the CX circuitry. Note that the grammar includes a special case for handling the string LRB in the 〈name〉 rule which corresponds to the left rubus (RB) region of CRE; this is necessary to prevent that string from being incorrectly parsed into LB (a string that does not correspond to any defined region) and RB.

Neuropils are denoted by the abbreviated names mentioned in Section 1; abbreviations corresponding to individual regions within each neuropil are detailed in the Supplementary Material. For neuropils that occur in pairs, upper case denotes the neuropil on the left side of the fly brain (from a dorsal perspective of the fly) while lower case denotes the neuropil on the right side of the fly brain. A neurite's type may be spine (s), bouton (or bleb) (b), or a combination thereof (bs, sb). In the absence of detailed data regarding synapses, information flow polarity is assumed to be reflected by neurite type; spines are assumed to be postsynaptic (and accept input), while boutons are assumed to be presynaptic (and emit output) (Wolff et al., 2015).

### 3.2. Executable circuit generation

To infer the presence of synaptic connections between neurons, each known biological neuron in the CX circuit was loaded into NeuroArch by name. A parser for the grammar described in Section 3.1 was used to extract records containing arborization information from each neuron's name (Table 1); these records were reinserted into the NeuroArch database as separate nodes connected to those that represent the original neurons.

**Table 1:** Fields in NeuroArch arborization data record.

**Field**	**Data type**	**Sample values**
neurite	set of “b” or “s”	[b], [b, s]
neuropil	string	PB, EB
region	set of strings or tuples	[L1], [(1, R1)]

*Region strings or tuples conform to the format described by the grammar in Section 3.1*.

After extraction of arborization data, all pairs of neurons in the database were compared to find those pairs with geometrically overlapping arborizations and differing neurite types (i.e., presynaptic vs. postsynaptic). This resulted in the creation of database nodes representing synapses that were connected to the associated biological neuron node pairs in NeuroArch's database.

To illustrate the synapse inference algorithm's operation, consider the neurons EB/([R3,R5],[P,M],[1-4])/s-EB/(R4,[P,M],[1-4])/b-LAL/RDG/b-PB/L3/b and PB/L4/s-EB/2/b-LAL/RVG/b. The former neuron has postsynaptic (spine) arborizations in EB and presynaptic (bouton) arborizations in EB and LAL; the latter has postsynaptic arborizations in PB and presynaptic arborizations in EB and LAL. Since the region EB/(R3,P,[1-4])/s in the former overlaps with region EB/2/b in the latter and the terminal types of the two neurons in the overlapping region differ, the presence of a synapse with information flow from the latter neuron to the former is inferred.

Although, physical overlap of arborizations does not always imply the presence of synapses, the above scheme illustrates how the software platform enables the use of partial structural information to construct and test CX circuit hypotheses. NeuroArch's data model can be extended to incorporate more detailed neural circuitry when it becomes available, thereby opening the doors to more accurate algorithmic inferences regarding the unknown portions of the CX circuit.

## 4. Results

### 4.1. Executable CX model response to visual input

In light of the current lack of data regarding synapses between the various neurons identified in the central complex neuropils, data regarding the arborizations of these neurons was used to infer the presence or absence of synapses to generate an executable model of the central complex. Local and projection neurons were assigned to neuropils as indicated in Table 2. The neuropils in which these neurons arborize and the terminal types of their arborizations is listed in Table 3. Further details regarding these neurons is included in the Supplementary Material.

**Table 2:** Assignment of neuron families to neuropils in generated CX model.

**Neuropil**	**Neuron families**
BU, bu	*BU-EB*
EB	EB-LAL-PB
FB	*FB local*
PB	PB local, PB-EB-NO, PB-EB-LAL, PB-FB-CRE, PB-FB-NO, PB-FB-LAL, WED-PS-PB, IB-LAL-PS-PB

*Arborization data for families in italics is hypothetical*.

**Table 3:** Identified neurons connecting CX and accessory neuropils.

**Neuron family**	**Locations of postsynaptic arborizations (dendrites)**	**Locations of presynaptic arborizations (axons)**	**References**
BU-EB	BU	EB	(Young and Armstrong, 2010b, p. 1509, Table 1)
EB-LAL-PB	EB	EB, LAL, PB	(Lin et al., 2013, Figures 4J–M)
FB local	FB	FB	(Young and Armstrong, 2010a, p. 1439)
IB-LAL-PS-PB	IB, LAL, PS	PB	(Lin et al., 2013, p. 1743, Figure 4A) (Wolff et al., 2015, Figure 3N)
PB local	PB	PB	(Lin et al., 2013, p. 1743),(Wolff et al., 2015, p. 1007)
PB-EB-LAL	PB	EB, LAL	(Lin et al., 2013, Figure 5E)
PB-EB-NO	PB	EB, NO	(Lin et al., 2013, p. 1745, Figure 5G)
PB-FB-CRE	PB	CRE, FB	(Lin et al., 2013, Figure 6F) (Wolff et al., 2015, Figure 3L)
PB-FB-LAL	PB	FB, LAL	(Lin et al., 2013, Figures 6F–H)
PB-FB-LAL-CRE	PB	CRE, FB, LAL	(Wolff et al., 2015, Figure 3M)
PB-FB-NO	PB	FB, NO	(Lin et al., 2013, p. 1746, Figure 5L)
PS-IB-PB	IB, PS	PB	(Wolff et al., 2015, Figures 3S,T)
PS-PB	PS	PB	(Wolff et al., 2015, Figure 3R)
WED-PS-PB	PS, WED	PB	(Lin et al., 2013, p. 1744, Figures 4B,D)

*Only neurons whose existence has been confirmed in (Young and Armstrong, 2010b; Lin et al., 2013; Wolff et al., 2015) are listed*.

**Figure 6:**
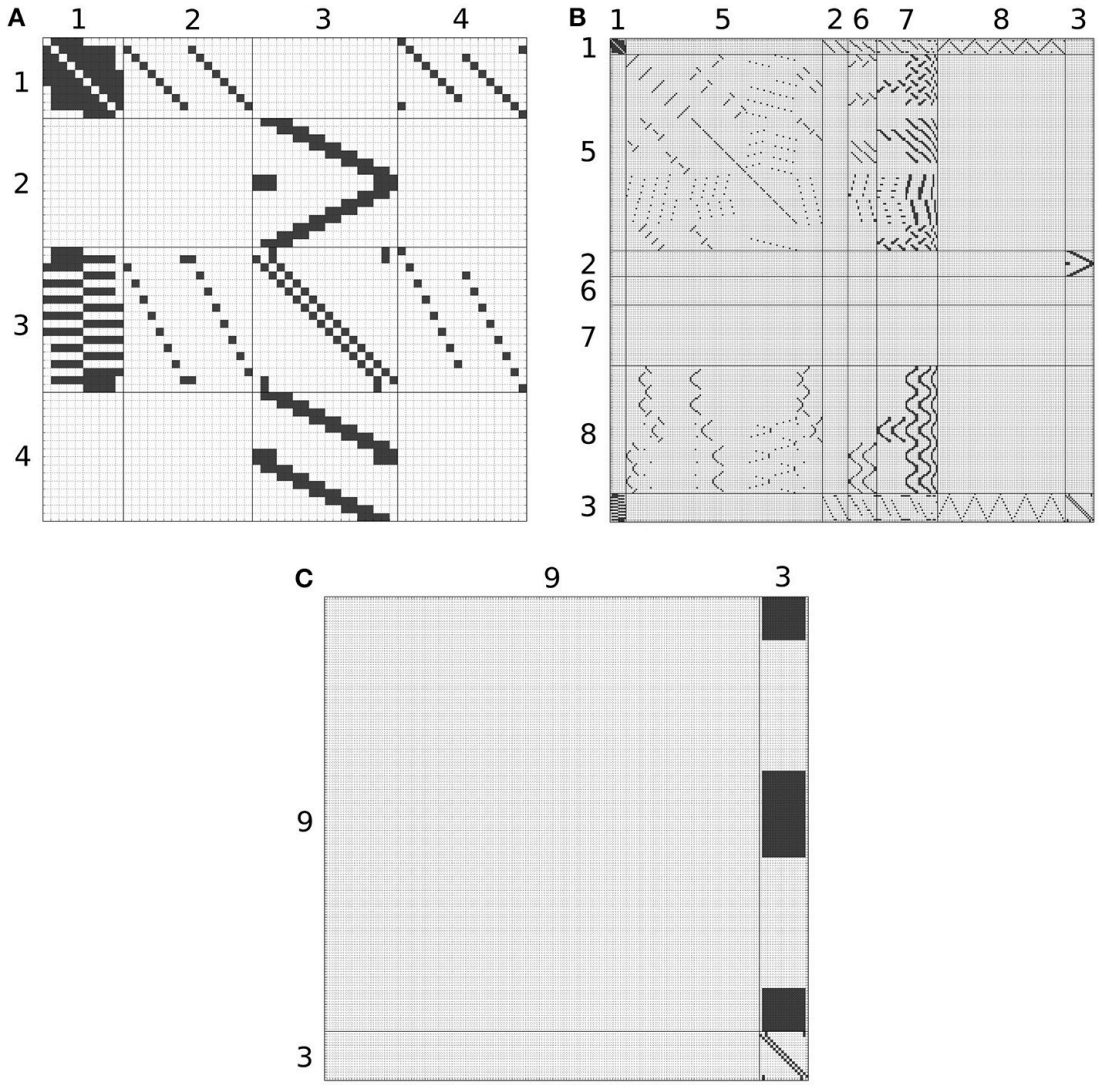
Inferred synapses between PB local (1), PB-EB-LAL (2), EB-LAL-PB (3), PB-EB-NO (4), FB local (5), PB-FB-CRE (6), PB-FB-LAL (7), PB-FB-NO (8), and BU-EB (9) neurons. Rows correspond to presynaptic neurons, while columns correspond to postsynaptic neurons. Owing to its size, the connectivity matrix is depicted as several overlapping matrices **(A–C)**.

Although, the BU-EB neurons have not been systematically characterized, available information regarding these neurons was used to hypothesize the arborization structure for a total of 80 BU-EB neurons in each hemisphere of the fly brain (Hanesch et al., 1989; Young and Armstrong, 2010b; Seelig and Jayaraman, 2013; Dewar et al., 2015). Likewise, we also hypothesized isomorphic sets of pontine neurons that link regions in FB based upon (Hanesch et al., 1989). The hypothesized arborizations of the BU-EB and pontine neurons were used to assign them names; the latter are detailed in the Supplementary Material. Figure 6 depicts the inferred synaptic connectivity between PB local, PB local, PB-EB-LAL, EB-LAL-PB, PB-EB-NO, FB local, PB-EB-LAL, PB-FB-CRE, PB-FB-LAL, PB-FB-NO, and BU-EB neurons; the rows of the connectivity matrix correspond to the presynaptic neurons, while the columns correspond to the postsynaptic neurons.

All neurons in the CX circuit were modeled as Leaky Integrate-and-Fire neurons, with the membrane voltage *V*_*i*_(*t*) of neuron *i* described by the differential equation

$$\begin{array}{l} {\dot{V}}_{i}\left(t\right)=-\frac{V_{i}\left(t\right)}{R_{i}C_{i}}+\frac{I_{i}\left(t\right)}{C_{i}} \end{array}$$

where *R*_*i*_ and *C*_*i*_ are the neuron's membrane resistance and capacitance and *I*_*i*_ the neuron's total input current. Upon reaching the threshold voltage *V*_*t, i*_, each neuron's membrane voltage is reset to *V*_*r, i*_. All synapses modeled to produce biexponential alpha function responses to presynaptic spikes; the synaptic conductance α_*i*_(*t*) = *ḡ*_*i*_*g*_*i*_(*t*) response to a spike at *t* = *t*_0_ is described by Ermentrout and Terman (2010)

$$\begin{array}{ll} {ġ}_{i}\left(t\right)= & h_{i}\left(t\right)u\left(t\right)\\ {ḣ}_{i}\left(t\right)= & -\left(a_{r,i}+a_{d,i}\right)h_{i}\left(t\right)-a_{r,i}a_{d,i}g_{i}\left(t\right)+\delta \left(t-t_{0}\right)a_{r,i}a_{d,i} \end{array}$$

where *ḡ*_*i*_ is the maximum conductance of the synapse, *u*(*t*) is the Heaviside step function, δ(*t*) the Dirac delta function, and *a*_*r, i*_ and *a*_*d, i*_ are the rise and decay time constants of the synapse's alpha function, respectively. The parameters of synapses between BU-EB neurons and other neurons were configured to exhibit inhibitory behavior; all remaining inferred synapses were configured to be excitatory. In all of the following connectivity matrices, a black square denotes the presence of a connection linking a presynaptic neuron on the y-axis to a postsynaptic neuron on the x-axis.

**Figure 7:**
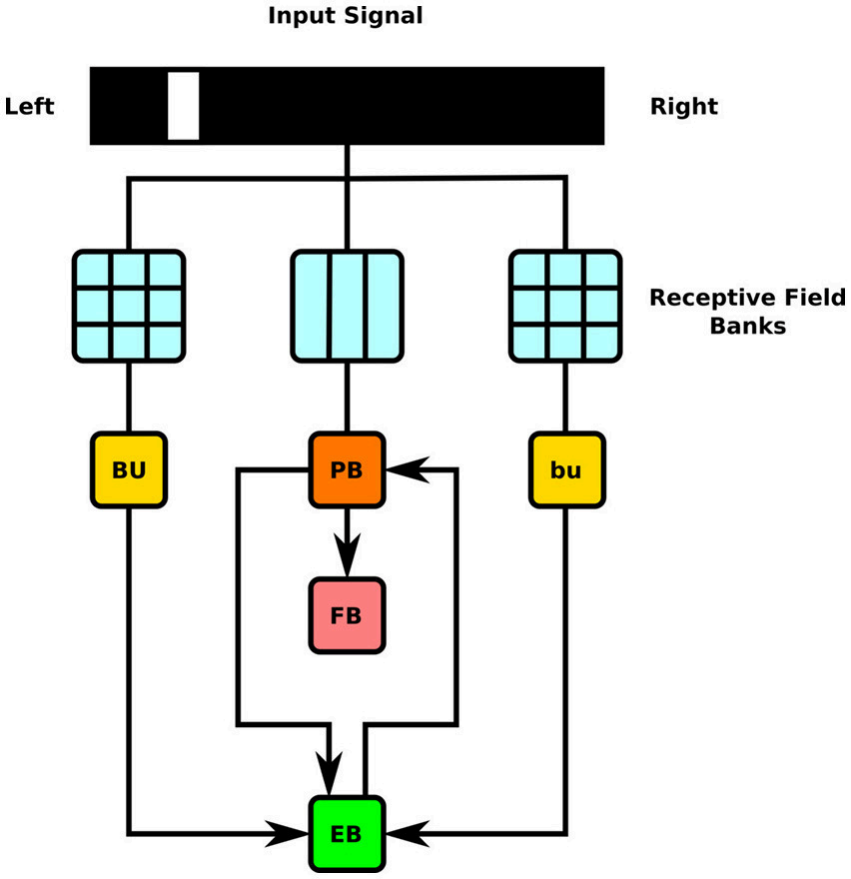
Schematic of information flow in generated CX model. 2D visual signals are passed through rectangular grids of Gaussian receptive fields whose outputs drive BU-EB neurons and through a bank of vertical rectangular receptive fields whose outputs drive neurons that innervate the PB glomeruli. The generated model only comprises neurons that innervate the depicted LPUs (BU, bu, EB, FB, and PB).

**Figure 8:**
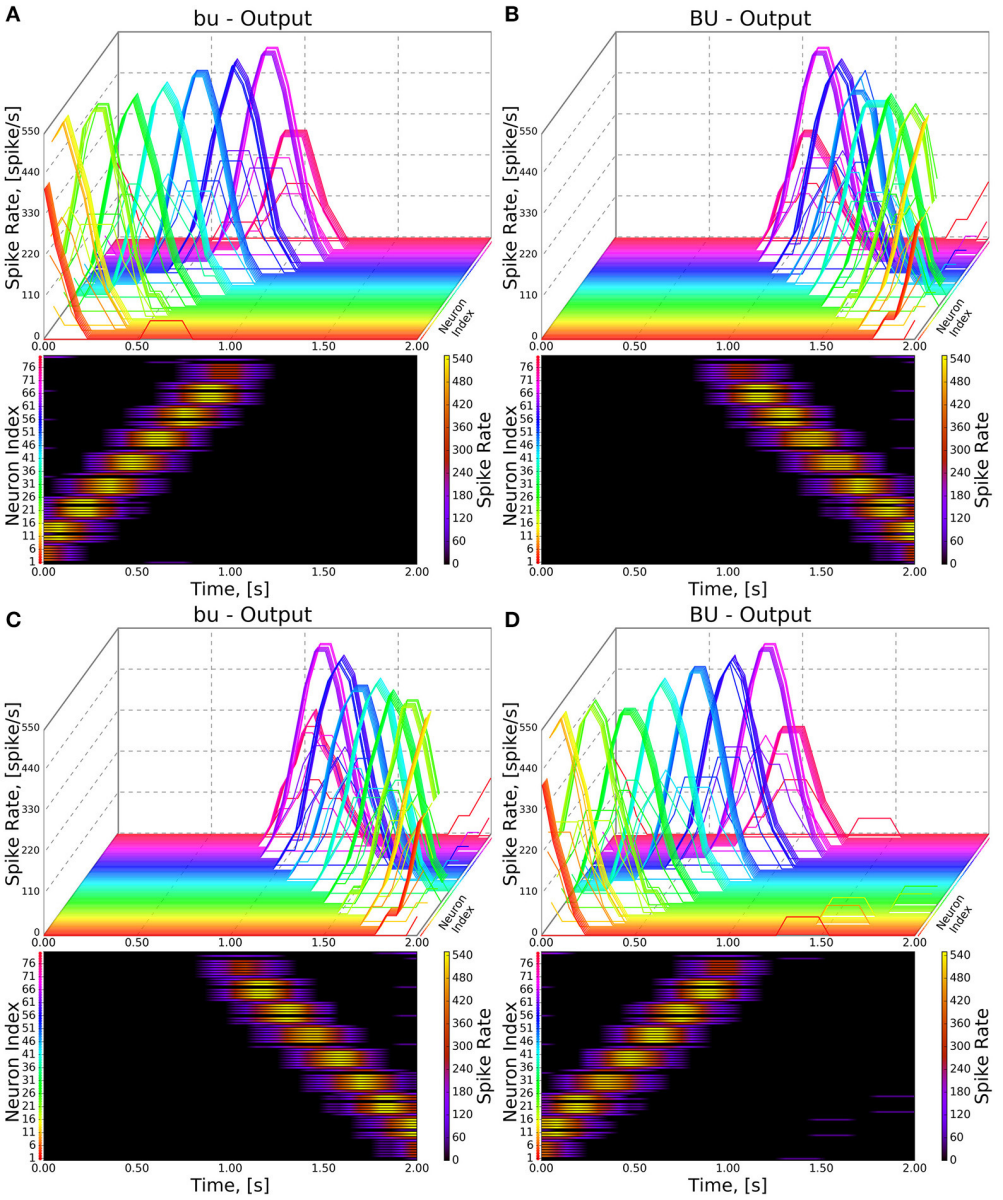
Response of CX projection neurons innervating BU/bu to moving bar input. (Top) The 3D view of the PSTH of a single neuron in the BU/bu family. Each line represents the PSTH of a single neuron. (Bottom) The heatmap view of the PSTH. Neurons are color coded with an one-to-one correspondence to the 3D view. Each row represents the PSTH of a single neuron (indicated by the color dot in front of each row). The PSTH was computed using a 200 ms bin size with a 50 ms sampling interval. **(A,B)** Response of bu/BU neurons to the left-to-right moving bar. **(C,D)** Response of bu/BU neurons to the right-to-left moving bar.

**Figure 9:**
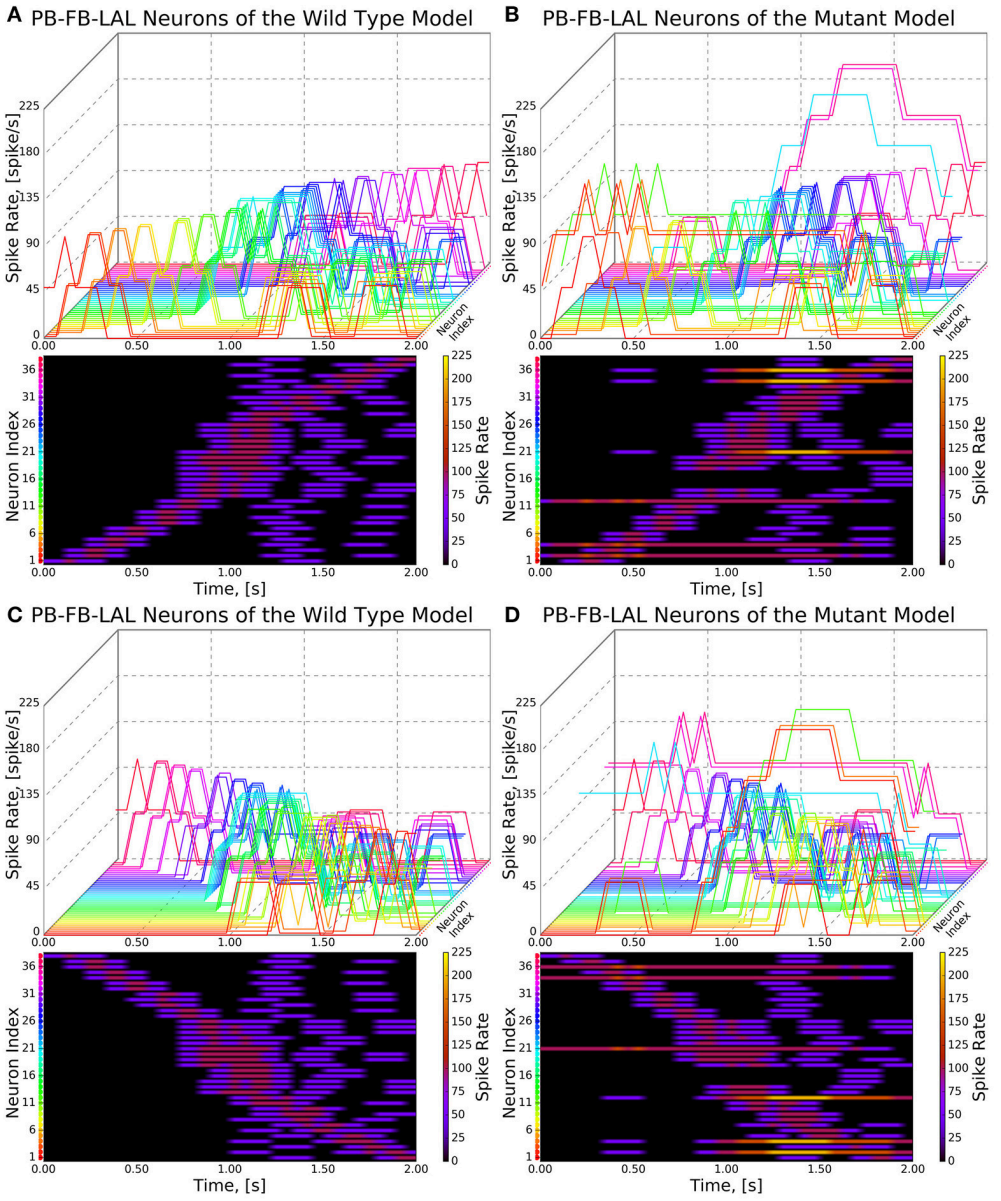
Response of CX projection neurons innervating PB to moving bar input (left to right). (Top) The 3D view of the peristimulus time histogram (PSTH) of a single neuron in the PB-FB-LAL family. Each line represents the PSTH of a single neuron. (Bottom) The heatmap view of the PSTH. Neurons are color coded with an one-to-one correspondence to the 3D view. Each row represents the PSTH of a single neuron (indicated by the color dot in front of each row). The PSTH was computed using a 200 ms bin size with a 50 ms sampling interval. **(A,C)** Response of the neurons in the wild type. **(B,D)** Response of the neurons in the *no bridge* mutant.

To test the executability of the generated circuit and its ability to respond to input data, the generated model was driven by a simple visual stimulus consisting of an illuminated vertical bar proceeding horizontally across the 2D visual space. Since the central complex neuropils do not receive direct connections from the vision neuropils, processing of the visual stimulus by the latter was approximated by three banks of receptive fields whose outputs were, respectively provided to BU, bu, and PB as input (Figure 7). In light of the reported retinotopy of bulb microglomeruli (Seelig and Jayaraman, 2013), the receptive fields for BU and bu were constructed as evenly spaced 2D grids of 80 circular Gaussians that respectively correspond to one of the microglomeruli in the bulb; each receptive field was connected to one BU-EB neuron such that the 16 neurons in each of the 5 groups of EB ring neurons processed input from a rectangle occupying $\frac{1}{5}$ of the 2D visual space. The azimuthal tracking of visual stimuli by activity in EB (Seelig and Jayaraman, 2015) and the mapping from the linear structure of PB to the circular structure of EB suggested that the PB glomeruli's receptive fields tile the fly's visual field; we therefore assigned 18 vertical rectangular regions with a constant magnitude to the respective glomeruli. Each receptive field was connected to all local and projection neurons that innervated the glomerulus corresponding to the receptive field region. The responses of the neurons in each family to the two input signals are organized in the same order in the respective raster plots. Figures 8, 9 depict the responses of neurons innervating the PB and BU/bu neuropils to an illuminated vertical bar moving from left to right across a dark background.

### 4.2. Comparing normal and abnormal neural circuits

To test hypotheses regarding incompletely characterized parts of the fly brain, one can create models that either attempt to replicate abnormal behaviors or emulate abnormal circuit structures observed in different mutant fly strains. For example, one can attempt to model phenotypes corresponding to mutations that constrict or disrupt connections between the left and right sides of PB such as *no bridge* and *tay bridge* by altering the PB model generation process accordingly. These mutations are known to alter the fly's step length and compromise the fly's directional targeting abilities (Triphan et al., 2010; Strauss, 2014). Since neurons innervating the motor ganglia are known to be postsynaptic to those that innervate LAL, it is reasonable to expect that analogous modifications to the structure of PB may alter the outputs of CX projection neurons that innervate LAL in a manner that reduces their sensitivity to directional visual stimuli.

**Figure 10:**
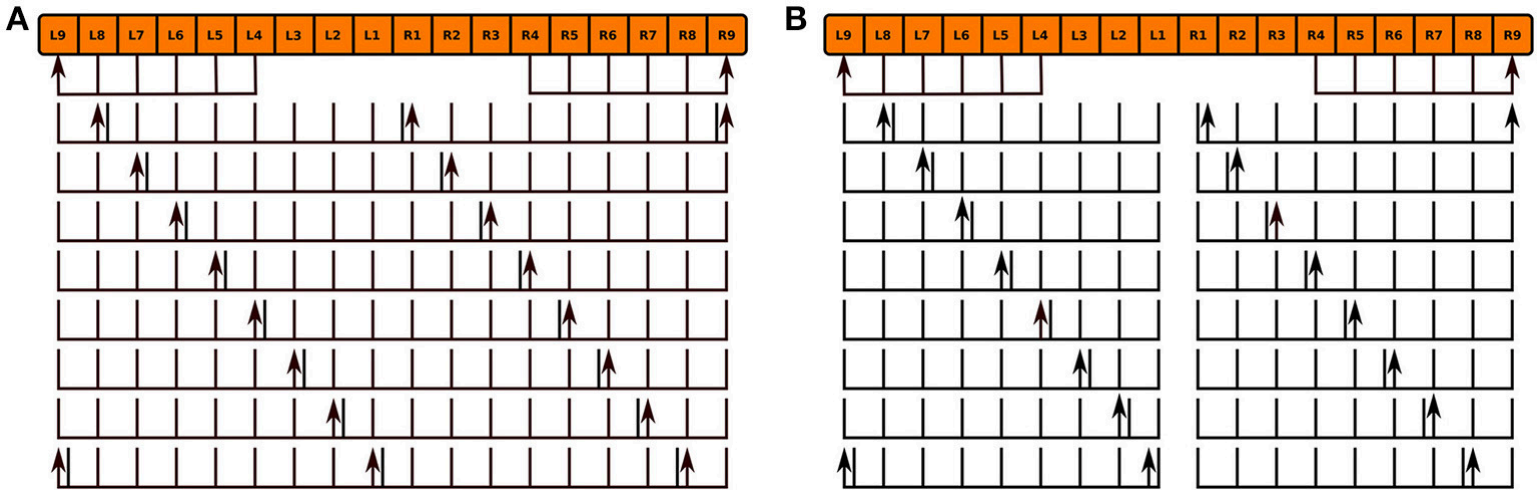
Normal PB local neuron innervation pattern **(A)** and hypothesized abnormal innervation pattern **(B)** in *no bridge* mutant. Arrows and lines respectively mark presynaptic and postsynaptic arborizations within the corresponding glomeruli; the presence of both presynaptic and postsynaptic arborizations within a glomerulus is marked by an adjacent arrow and line.

We modeled the *no bridge* mutant by positing the development of 16 PB local neurons that only span either the left or right sides of PB in place of the 8 local neurons that normally span the entire neuropil in the wild type fly (Figure 10; the hypothesized neurons' names are listed in the Supplementary Material). Although, observations of the *no bridge* mutant suggest that several of the medial glomeruli are not present, this model does not alter any of the other known neurons in CX. The synapse inference algorithm (Section 3.2) was then run on the modified circuits to construct a mutant CX model.

As the inputs to the wild type and mutant models are identical and the BU-EB neurons do not receive any input from other neurons in the generated model, their responses in the mutant model are identical to those in the wild type model (Figure 8). The effects of the mutation on the response of the PB-FB-LAL projection neurons can be observed by comparing the mutant model output in Figure 9B to Figure 9A; in both cases, the PB-FB-LAL neurons along the vertical axes of the PSTH plots are arranged from those that innervate the leftmost glomerulus to the rightmost glomerulus. The PB-FB-LAL neurons in the wild type model exhibit sensitivity to the direction of the visual stimulus across the azimuth. While some of this activity occurs in the mutant model, the mutation causes three PB-FB-LAL neurons on the left and three on the right sides of the fly's brain to produce high activity over abnormally long stretches of time. We posit that the output of these neurons may dominate the inputs to the LAL neuropils and effectively drown out the directionally sensitive responses of the other PB-FB-LAL neurons. This could explain the inability of fly mutants with a laterally interrupted PB to perform the directional targeting necessary to successfully traverse gaps in climbing experiments (Triphan et al., 2010).

## 5. Conclusion

Kakaria and De Bivort (2017) describe a model of the PB and EB circuitry that exhibit ring attractor dynamics similar to those observed in calcium imaging of EB responses to visual landmark stimuli (Seelig and Jayaraman, 2015). While this model comprises the same neuron families as generated model described in Section 4.1, the synaptic connections inferred in Figure 6 for those neuron families used in both models differ owing to our incorporation of arborization information from both Lin et al. (2013) and Wolff et al. (2015) rather than the latter alone. Our model also incorporates neuron families that innervate FB.

We have demonstrated how NeuroGFX enables the structure of the CX neuropils to be probed simultaneously with execution of neural circuit models inferred from available connectomic data. Although, the NeuroArch component of our software supports extensive customization of supported executable circuit components, the software's current web interface is read-only. We are extending this interface to enable users to directly manipulate the executed circuit by defining new modeling components, loading alternative subcircuits into NeuroArch for evaluation, and modifying the parameters of stored circuit models.

Assessment of CX model accuracy requires a means of analyzing its response to different input signals. Since the CX circuit comprises multiple putative input and output pathways of interest, there is a need to support concurrent injection of inputs and recording of responses from potentially any component in a circuit model. While models of sensory neuropils can be analyzed using prerecorded or generated sensory inputs, similar analysis of non-sensory neuropil models requires the ability to observe their behavior when they receive input from models of sensory neuropils. The communication interface described in Section 2 that Neurokernel and NeuroArch support to enable the integration of models of different neuropils already provides the requisite internal functionality to both inject and record either analog or spike signals into specific model components. We will extend the NeuroGFX interface to enable users to provide prerecorded input signals for injection into the CX circuit and designate which circuit components to stimulate. We also will extend the FFBO component of our software to explicitly support future web applications that let users link CX models to those of other neuropils in the fly's brain.

We aim to incorporate more detailed connectomic data into the application's NeuroArch database; ongoing work by the developers of the FlyCircuit database (Chiang et al., 2011) that utilizes neuron morphology to infer the number of synapses between neurons will enable construction of CX models with more accurate connectivity patterns than those currently inferred from arborization overlap^1^.

## Author contributions

AL initiated the project and critically reviewed the manuscript text and figures. LG and CY developed the executable CX models. LG developed the NeuroArch/Neurokernel software used for model representation/execution, wrote the manuscript, and prepared the supplementary circuit figures and tables. CY developed the NeuroGFX/FFBO software for CX visualization, prepared the manuscript figures, and reviewed the manuscript.

### Conflict of interest statement

The authors declare that the research was conducted in the absence of any commercial or financial relationships that could be construed as a potential conflict of interest.

## Footnotes

^1^C.C. Lo, private communication.
