# Supplementary material for: Generating Executable Models of the Drosophila Central Complex
Source: Front Behav Neurosci. 2017 May 30;11:102. doi: 10.3389/fnbeh.2017.00102 (PMC5447672; doi:10.3389/fnbeh.2017.00102)
Supplement: Supplementary file 1 [file Presentation1.PDF]

Supplementary Material: Generating Executable Models of the Drosophila Central Complex

Lev E. Givon [1], Aurel A. Lazar [2,*] and Chung-Heng Yeh [2]
*Correspondence:
Aurel A. Lazar
aurel@ee.columbia.edu

1 NEUROPIL NOMENCLATURE

Drosophila neuropils are identified using the names in Tab. S1; this nomenclature is based upon that described in (Ito et al., 2014). Some neuropils are referred to by different names either in other literature or in other insects; (Ito et al., 2014, Tab. S13) maps the employed nomenclature to that used in (Chiang et al., 2011) and - for the most part - in (Lin et al., 2013).

2 STRUCTURE OF NEUROPILS IN AND ASSOCIATED WITH THE CENTRAL COMPLEX

This section presents details regarding the high-level structure of the various CX neuropils and the accessory neuropils to which they are connected.

2.1 Protocerebral Bridge (PB)

The PB neuropil comprises 18 regions called glomeruli (Wolff et al., 2015) connected to other substructures within the CX (Fig. S1). The local neuron population of the PB comprises 8 (Wolff et al., 2015, p. 1007) or 10 (Lin et al., 2013, p. 1743) types of local neurons (Fig. S8, Tab. S3). A single PB region label matches the following regular expression:

$$\langle \text{glomerulus} \rangle := [\text{L}, \text{R}][1-9]$$

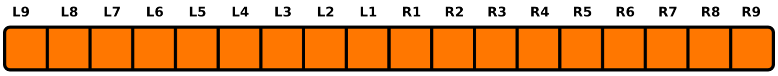

Figure S1. Schematic of regions in PB used to identify neurons by their arbors (Lin et al., 2013; Wolff et al., 2015).

Abbreviation	Name	Notes
AOTU	Anterior optic tubercle	Also known as optic tubercle (OPTU (Chiang et al., 2011)).
ATL	Antler	Corresponds to dorsal part of caudalcentral protocerebrum (CCP) (Ito et al., 2014, Tab. S13).
BU	Bulb	Also known as lateral triangle (LT, LAT, Lat Tri (Lin et al., 2013, Tab. S13) or LTR (Hanesch et al., 1989)).
CRE	Crepine	Posterior part also known as dorsal part of IDFP (Ito et al., 2014, Tab. S13); comprises a region called the rubus (RUB) (Wolff et al., 2015, p. 1001) or round body (RB) (Wolff et al., 2015, p. 1031).
LAL	Lateral accessory lobe	Also known ventral body (VBO (Hanesch et al., 1989)) or inferior dorsofrontal protocerebrum (IDFP) (Lin et al., 2013, Fig. S1). Comprises the gall (GA) (Ito et al., 2014, Tab. S13), whose dorsal and ventral portions are referred to as the dorsal and ventral spindle bodies (DSB and VSB, respectively) (Wolff et al., 2015, p. 1021).
EB	Ellipsoid body	Also known as lower central body (CBL (Pfeiffer and Homberg, 2014)).
FB	Fan-shaped body	Also known as upper central body (CBU (Pfeiffer and Homberg, 2014)).
IB	Inferior Bridge (IB)	Corresponds to ventral part of caudalcentral protocerebrum (CCP) (Ito et al., 2014, Tab. S13).
LO	Lobula	
LOP	Lobula Plate	
NO	Noduli	
PS	Posterior slope	Corresponds to caudalmedial protocerebrum (CMP) and - possibly - part of the ventromedial protocerebrum (VMP) (Ito et al., 2014, Tab. S13).
PB	Protocerebral Bridge	
SMP	Superior medial protocerebrum	Corresponds to superior dorsolateral protocerebrum (SDFP) and medial part of inner dorsolateral protocerebrum. (IDLP) (Ito et al., 2014, Tab. S13).
VLP	Ventrolateral protocerebrum	Contains optic glomeruli (Ito et al., 2014, p. 42, Supp.).
WED	Wedge	Also known as the caudal ventrolateral protocerebrum (CVLP) (Ito et al., 2014, p. 42, Supp.).

Table S1. Neuropil names.

9 2.2 Fan-Shaped Body (FB)

The FB neuropil comprises multiple lateral layers (Pfeiffer and Homberg, 2014); most recent work suggests the presence of 9 layers (Wolff et al., 2015, p. 1011). The neuropil is subdivided vertically into 8 (Lin et al., 2013) or 7 (Wolff et al., 2015, p. 1010) columns called segments (Hanesch et al., 1989); however, it seems that only some of its layers (1-5) exhibit clearly columnar structure (Wolff et al., 2015, p. 1008) (S2). Regions in FB are connected by local neurons called *pontine neurons*; some of these neurons connect adjacent layers, while others connect adjacent segments (Hanesch et al., 1989, p. 349, 352). A representative class of pontine neurons comprising symmetric neurons that connect each segment in one side of FB with each segment in the other side such that the presynaptic and postsynaptic arborizations are 4 segments apart (Hanesch et al., 1989, p. 352) (although more recent work suggests that each neuron might be a bundle of 2 neurons (Young and Armstrong, 2010a, p. 1439)) is depicted in Tab. S4. Other classes dorsoventrally connecting different layers in FB may exist, but they have not been systematically identified. A single region in FB is denoted by a label matching the regular expression

$$\langle \text{region} \rangle := \backslash(\overbrace{[1-9]}^{\text{layer}}, \overbrace{[\mathrm{L}, \mathrm{R}][1-4]}^{\text{segment}}\backslash)$$

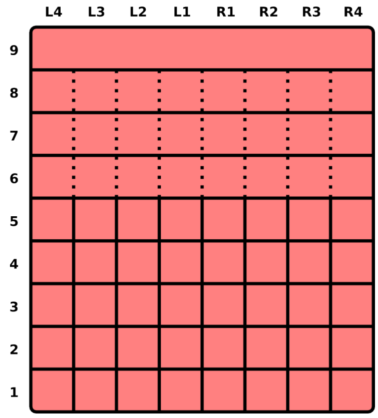

Figure S2. Schematic of regions in FB used to identify neurons by their arbors (Lin et al., 2013; Wolff et al., 2015).

2.3 Ellipsoid Body (EB)

The EB neuropil is a toroidal structure that comprises 16 wedges (Wolff et al., 2015, p. 1013) (Fig. 3a), 8 tiles (Wolff et al., 2015, p. 1018) (Fig. 3b), 3 shells (anterior, medial, posterior) (Wolff et al., 2015, p. 1013), and 4 rings (Lin et al., 2013) (Fig. 3c). Wedges extend radially through full radius of the EB torus and occupy the posterior and medial shells or all 3 shells (Wolff et al., 2015, p. 1013). Tiles are restricted to the posterior shell (Wolff et al., 2015, p. 1014); tiles geometrically overlap with corresponding wedges as described in Tab. S2. Although EB appears to contain local neurons (Chiang et al., 2011), these neurons have not yet been systematically identified; there is some evidence for EB pontine neurons in related fly species such as *Neobellieria* (Phillips-Portillo, 2012, p. 11). Each region in EB is denoted by a label that matches the regular expression

$$\langle\text{region}\rangle := \overbrace{[1-8]}^{\text{tile}} \,|\, \backslash(\overbrace{[\mathrm{L},\mathrm{R}][1-8]}^{\text{wedge}}, \overbrace{[\mathrm{P},\mathrm{M},\mathrm{A}]+}^{\text{shell}}, \overbrace{[1-4]}^{\text{ring}} \backslash)$$

For EB regions other than tiles, the region denoted by a label comprises the volume intersected by the specified wedges, shells, and rings. For example, $(\mathrm{L1}, [\mathrm{P}, \mathrm{M}], 4)$ represents the volume in which wedge $\mathrm{L1}$, shells P and M, and ring 4 overlap.

Tile	Wedge
EB/1/x	EB/([L1,R1],P,x)/x
EB/2/x	EB/(R[2,3],P,x)/x
EB/3/x	EB/(R[4,5],P,x)/x
EB/4/x	EB/(R[6,7],P,x)/x
EB/5/x	EB/([R8,L8],P,x)/x
EB/6/x	EB/(L[6,7],P,x)/x
EB/7/x	EB/(L[4,5],P,x)/x
EB/8/x	EB/(L[2,3],P,x)/x

Table S2. Geometric overlap between EB tiles and wedges.

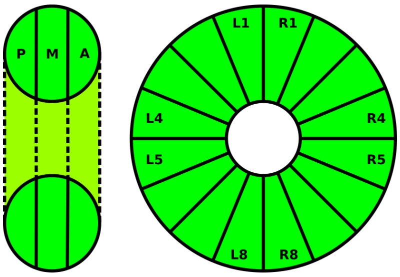

(3a) Shells (L) and wedges (R)(Wolff et al., 2015).

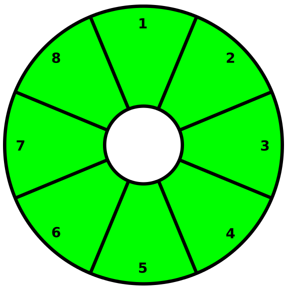

(3b) Tiles (Wolff et al., 2015).

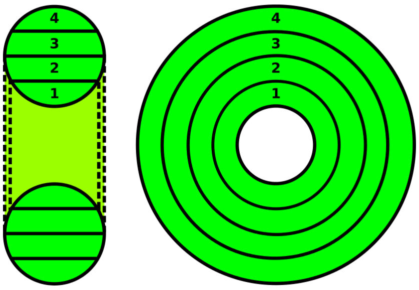

(3c) Rings (Young and Armstrong, 2010b).

Figure S3. Schematics of regions in EB. All circular schematics are anterior; sagittal views in Figs. 3a and 3c are posterior to anterior from left to right).

2.4 Noduli (NO)

The NO neuropils comprise 3 distinct structures (NO1, NO2, NO3) divided into subcompartments (Fig. S4) (Wolff et al., 2015, p. 1017). The noduli lack segregated populations of local neurons (Chiang et al., 2011, p. 5). Each NO region label matches the following regular expressions:

$$\langle \text{subcompartment} \rangle := \begin{cases} [\text{L}, \text{R}] & \text{for NO1} \\ [\text{L}, \text{R}][\text{V}, \text{D}] & \text{for NO2} \\ [\text{L}, \text{R}][\text{A}, \text{M}, \text{P}] & \text{for NO3} \end{cases}$$

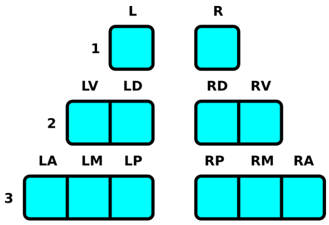

Figure S4. Schematic of regions in NO used to identify neurons by their arbors (Wolff et al., 2015).

2.5 Bulb (BU)

Each of the BU neuropils comprises multiple regions referred to as microglomeruli. There appear to be 80 microglomeruli in each BU neuropil (Fig. S5) (Lin et al., 2013, p. 1741). These microglomeruli ostensibly exhibit retinotopic organization (Seelig and Jayaraman, 2013). Each of the BU region labels matches the regular expression

$$\langle \text{microglomerulus} \rangle := [\text{L}, \text{R}][0-9]+$$

where the integer portion of the labels ranges from 1 to 80.

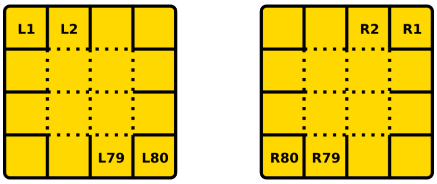

Figure S5. Schematic of regions in BU used to identify neurons by their arbors (Lin et al., 2013). The relative positions of the regions does not necessarily correspond to their actual physical positions.

2.6 Lateral Accessory Lobe (LAL)

Each LAL neuropil comprises a region called the gall that is subdivided into a tip, dorsal, and ventral subregion; the remainder of LAL is referred to as the hammer body (HB) (Fig. S6) (Lin et al., 2013). Each of these regions has a label that matches the regular expression

$$\langle \text{region} \rangle := [\text{L}, \text{R}]\,(\text{HB}|\text{GT}|\text{DG}|\text{VG})$$

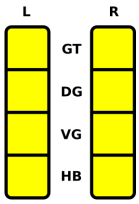

Figure S6. Schematic of regions in LAL used to identify neurons by their arbors (Wolff et al., 2015).

2.7 Crepine (CRE)

Each CRE neuropil is divided into two regions that respectively correspond to the rubus (RB) and remainder of the crepine (CRE) (Fig. S7); these match the regular expression

$$\langle \text{region} \rangle = [\mathrm{L}, \mathrm{R}]\,(\mathrm{RB}|\mathrm{CRE})$$

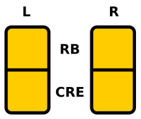

Figure S7. Schematic of regions in CRE used to identify neurons by their arbors (Wolff et al., 2015).

2.8 Other Neuropils (IB, PS, SMP, WED)

Distinct regions of interest within IB, PS, SMP, and WED have not been identified; they are therefore regarded as comprising single regions on each side of the fly brain. Each region in these neuropils matches the regular expression

$$\langle \text{region} \rangle := [\text{L}, \text{R}]\ (\text{IB}|\text{PS}|\text{SMP}|\text{WED})$$

3 IDENTIFIED NEURONS IN THE CENTRAL COMPLEX

In all neuropil innervation diagrams depicted below, arrow heads represent presynaptic arborizations and arrow tails represent postsynaptic arborizations.

3.1 Local Neurons

3.1.1 PB Local Neurons

Different studies of PB have identified 8 (Wolff et al., 2015, p. 1007) or 10 (Lin et al., 2013, p. 1743) distinct local neurons. Tab. S3 and Fig. S8 assume the presence of 8 glomeruli on each side of PB as indicated by (Wolff et al., 2015), that R2-R9 in (Wolff et al., 2015) correspond to R1-R8 in (Lin et al., 2013), and that postsynaptic arborizations are spaced 7 glomeruli apart in all but the first 2 neuron types.

<table>
<tr><th></th><th>Label</th></tr>
<tr><td>1</td><td>PB/L9/b-PB/L[4-8]/s</td></tr>
<tr><td>2</td><td>PB/L8|R1|R9/b-PB/L[1-9]|R[1-9]/s</td></tr>
<tr><td>3</td><td>PB/L7|R2/b-PB/L[1-9]|R[1-9]/s</td></tr>
<tr><td>4</td><td>PB/L6|R3/b-PB/L[1-9]|R[1-9]/s</td></tr>
<tr><td>5</td><td>PB/L5|R4/b-PB/L[1-9]|R[1-9]/s</td></tr>
<tr><td>6</td><td>PB/R5|L4/b-PB/R[1-9]|L[1-9]/s</td></tr>
<tr><td>7</td><td>PB/R6|L3/b-PB/R[1-9]|L[1-9]/s</td></tr>
<tr><td>8</td><td>PB/R7|L2/b-PB/R[1-9]|L[1-9]/s</td></tr>
<tr><td>9</td><td>PB/R8|L1|L9/b-PB/R[1-9]|L[1-9]/s</td></tr>
<tr><td>10</td><td>PB/R9/b-PB/R[4-8]/s</td></tr>
</table>

Table S3. PB local neurons.

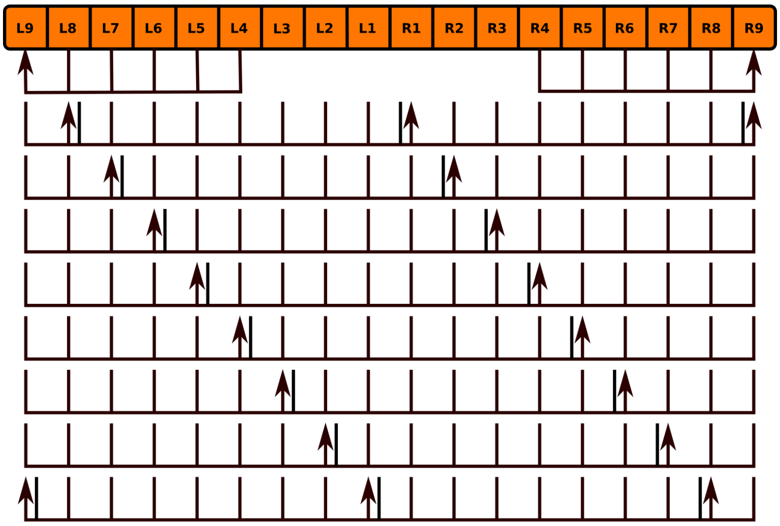

Figure S8. Innervation pattern of PB local neurons (Tab. S3).

3.1.2 FB Local Neurons

Several classes of local neurons referred to as pontine neurons have been observed to connect different regions of FB with each other (Hanesch et al., 1989, p. 349), (Young and Armstrong, 2010b, p. 1507). One class (Tab. S4) comprises symmetric neurons that connect each segment on one side of the FB with each segment on the other side such that the presynaptic and postsynaptic arborizations are 4 segments apart (Hanesch et al., 1989, p. 352) (although more recent work suggests that each neuron might be a bundle of 2 neurons (Young and Armstrong, 2010a, p. 1439)). Judging by the structure of pontine neurons in other insects (Heinze and Homberg, 2008), arborizations might not be strictly confined to targeted regions. Other classes dorsoventrally connecting different layers in FB may exist, but they have not been systematically identified (Hanesch et al., 1989, p. 349). It is unclear whether local neurons other than pontine neurons exist in the FB.

	**Label**
1	FB/(3,L4)/s-FB/(3,R1)/b
2	FB/(3,L3)/s-FB/(3,R2)/b
3	FB/(3,L2)/s-FB/(3,R3)/b
4	FB/(3,L1)/s-FB/(3,R4)/b
5	FB/(3,R4)/s-FB/(3,L1)/b
6	FB/(3,R3)/s-FB/(3,L2)/b
7	FB/(3,R2)/s-FB/(3,L3)/b
8	FB/(3,R1)/s-FB/(3,L4)/b

Table S4. One identified class of FB local neurons.

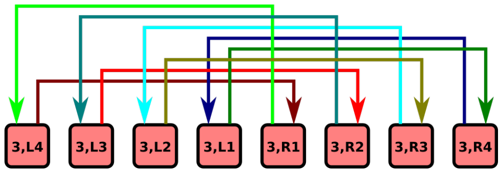

Figure S9. Innervation pattern of FB local neurons (Tab. S4).

3.1.3 EB Local Neurons

Although there appear to be local neurons in EB (Chiang et al., 2011), they do not appear to have been systematically identified yet.

3.2 Projection Neurons

3.2.1 BU-EB Projection Neurons

Neurons with postsynaptic arborizations in BU and presynaptic arborizations in EB are typically referred to as ring or R neurons (Hanesch et al., 1989, p. 352) by virtue of the shape of their EB arborizations. 5 types of ring neurons (R1, R2, R3, R4m, R4d) have been observed (Young and Armstrong, 2010b, p. 1509); specific ring neuron types appear to be essential to different visual behaviors (Dewar et al., 2015, p. 120). Each ring neuron type arborizes in a single microglomerulus (Seelig and Jayaraman, 2013, p. 262) and a different portion of the EB radius (Fig. S10); these types correspond to different sets of BU microglomeruli (and hence comprise multiple neurons). About 20 of each of these types of neurons have been estimated in each hemisphere of the fruit fly brain (Young and Armstrong, 2010b, p. 1510); combined with visual confirmation of the presence of 80 microglomeruli (§ 2.5), this suggests that there are 16 of each neuron type present in BU. Some ring neurons are GABAergic (Lin et al., 2013, p. 1750), while others are glutamatergic (Lin et al., 2013, Fig. 7C); their synaptic connections to other neurons in EB therefore seem to be inhibitory. There is recent evidence that some ring neurons may be cholinergic and hence possess excitatory synapses (Martín-Peña et al., 2014, p. 1598). Coincident synapses (i.e., those in which two independent presynaptic zones coincide with a single postsynaptic zone) have been observed in EB between ring neurons in specific domains and other neurons both in and outside of those domains. (Martín-Peña et al., 2014, p. 1592); it seems that such synapses may also exist between other neurons that innervate EB (Martín-Peña et al., 2014, p. 1594). Connections between AOTU and BU have been observed (Otsuna et al., 2014, p. 9); these presumably constitute a pathway for input visual information from LO via AOTU (Otsuna and Ito, 2006, p. 939) to EB via ring neurons.

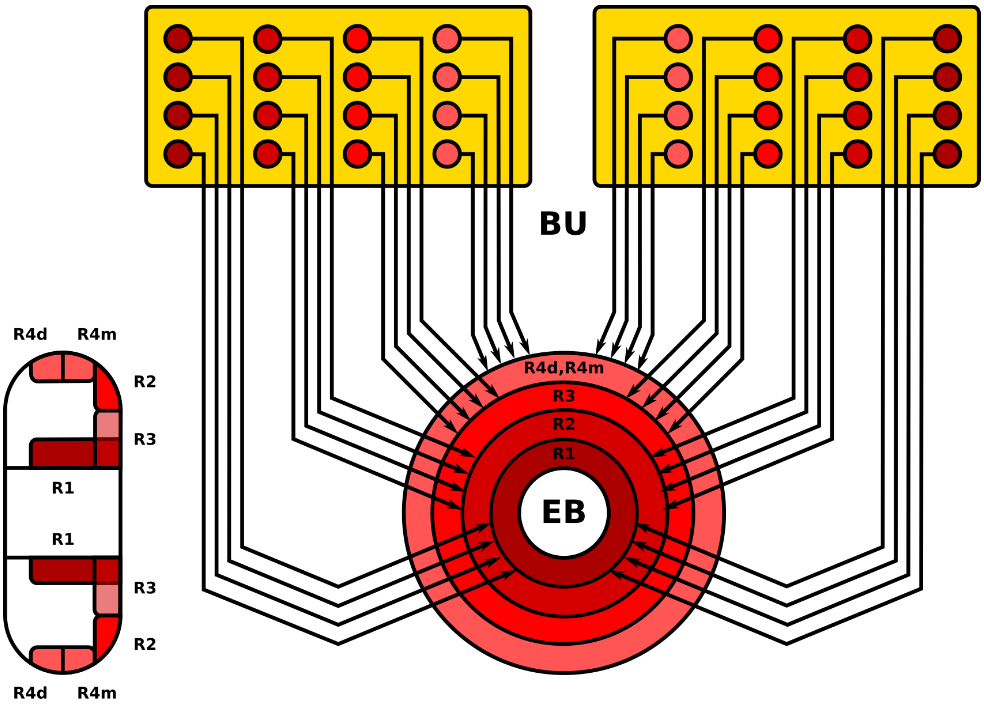

Figure S10. Spatial organization of ring neuron arborizations in BU microglomeruli (yellow) and regions in EB (red); posterior is left of sagittal section of EB, anterior is right. The depicted EB regions do not exactly correspond to the rings in Fig. 3c. Only a fraction of the microglomeruli/neurons are depicted. The spatial organization of each neuron type's microglomeruli is unclear.

3.2.2 EB-FB-LAL-SMP Projection Neurons

Neurons with presynaptic ring-shaped arborizations in EB and postsynaptic arborizations in other neuropils are referred to as extrinsic ring neurons. Two types (ExR1, ExR2) have been observed; these neurons appear to constitute a dopaminergic pathway (Lin et al., 2013, Fig. 7C). ExR1 neurons are presynaptic in EB, FB, and LAL, and postsynaptic in SMP (Hanesch et al., 1989, p. 353) [VirtualFlyBrain]. ExR2 appears to have presynaptic arborizations in EB and arborizations in SMP, but the remainder of the neuron's structure has not been reconstructed.

3.2.3 EB-LAL-PB Projection Neurons

These neurons correspond to the EB-PB-VBO or EIP neurons in (Lin et al., 2013, Fig. 2); they constitute a cholinergic pathway (Lin et al., 2013, Fig. 7C). The neuron arborizations in Tab. S5 and Fig. S11 make the assumption that the C, O, and P rings in (Lin et al., 2013) collectively correspond to the P and M shells in (Wolff et al., 2015) and that the C and P rings collectively correspond to the P shell.

	Label
1	EB/(L8,P,[1-4])/s-EB/(L8,P,[1-4])/b-lal/RDG/b-PB/L9/b
2	EB/([L7,R8],[P,M],[1-4])/s-EB/(L8,[P,M],[1-4])/b-LAL/LDG/b-PB/R1|L1/b
3	EB/([L8,L6],[P,M],[1-4])/s-EB/(L7,[P,M],[1-4])/b-lal/RVG/b-PB/L8/b
4	EB/([L5,L7],[P,M],[1-4])/s-EB/(L6,[P,M],[1-4])/b-LAL/LVG/b-PB/R2/b
5	EB/([L6,L4],[P,M],[1-4])/s-EB/(L5,[P,M],[1-4])/b-lal/RDG/b-PB/L7/b
6	EB/([L3,L5],[P,M],[1-4])/s-EB/(L4,[P,M],[1-4])/b-LAL/LDG/b-PB/R3/b
7	EB/([L4,L2],[P,M],[1-4])/s-EB/(L3,[P,M],[1-4])/b-lal/RVG/b-PB/L6/b
8	EB/([L1,L3],[P,M],[1-4])/s-EB/(L2,[P,M],[1-4])/b-LAL/LVG/b-PB/R4/b
9	EB/([L2,R1],[P,M],[1-4])/s-EB/(L1,[P,M],[1-4])/b-lal/RDG/b-PB/L5/b
10	EB/([R2,L1],[P,M],[1-4])/s-EB/(R1,[P,M],[1-4])/b-LAL/LDG/b-PB/R5/b
11	EB/([R1,R3],[P,M],[1-4])/s-EB/(R2,[P,M],[1-4])/b-lal/RVG/b-PB/L4/b
12	EB/([R4,R2],[P,M],[1-4])/s-EB/(R3,[P,M],[1-4])/b-LAL/LVG/b-PB/R6/b
13	EB/([R3,R5],[P,M],[1-4])/s-EB/(R4,[P,M],[1-4])/b-lal/RDG/b-PB/L3/b
14	EB/([R6,R4],[P,M],[1-4])/s-EB/(R5,[P,M],[1-4])/b-LAL/LDG/b-PB/R7/b
15	EB/([R5,R7],[P,M],[1-4])/s-EB/(R6,[P,M],[1-4])/b-lal/RVG/b-PB/L2/b
16	EB/([R8,R6],[P,M],[1-4])/s-EB/(R7,[P,M],[1-4])/b-LAL/LVG/b-PB/R8/b
17	EB/([R7,L8],[P,M],[1-4])/s-EB/(R8,[P,M],[1-4])/b-lal/RDG/b-PB/L1|R1/b
18	EB/(R8,P,[1-4])/s-EB/(R8,P,[1-4])/b-LAL/LDG/b-PB/R9/b

Table S5. EB-LAL-PB neurons.

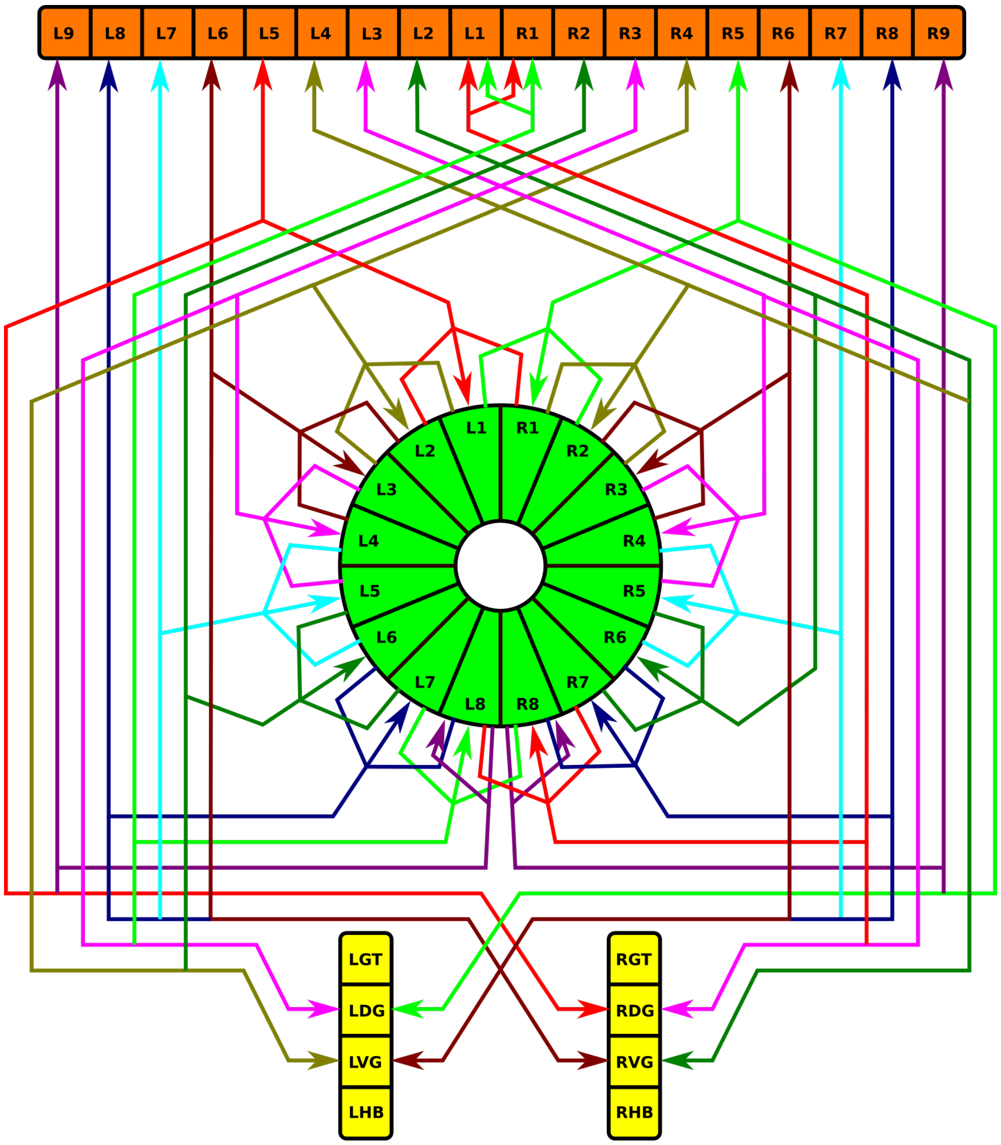

Figure S11. Neuropil innervation pattern for EB-LAL-PB neurons (Tab. S5).

3.2.4 F Projection Neurons

F neurons innervate entire layers of FB, with some types associated with specific layers (Hanesch et al., 1989, p. 353). Fm neurons have bleb-like (presynaptic) arborizations in layer 2 of FB; Fm1 neurons have spiny (postsynaptic arborizations in SLP or SIP, Fm2 neurons have spiny arborizations in LAL, and Fm3 neurons have spiny arborizations in ICL (Hanesch et al., 1989, p. 353). Some Fl neurons have spiny branches in LAL (Hanesch et al., 1989, p. 354), while other Fl neurons have spiny branches that innervate the entire BU (Hanesch et al., 1989, p. 354).

3.2.5 IB-LAL-PS-PB Projection Neurons

These neurons corresponds to the CVLP-IDFP-VMP-PB or CIVP neurons in (Lin et al., 2013, Fig. 2) and the PB-LAL-PS neurons in (Wolff et al., 2015, Fig. 3N). They receive indirect input from the vision system and innervate all glomeruli in PB indicated in Tab. S6:

	**Label**
1	IB/L/s-LAL/LHB/s-PS/L/s-PB/L[2-9]|R[2-9]/b
2	ib/R/s-lal/RHB/s-ps/R/s-PB/L[2-9]|R[2-9]/b

Table S6. IB-LAL-PS-PB neurons.

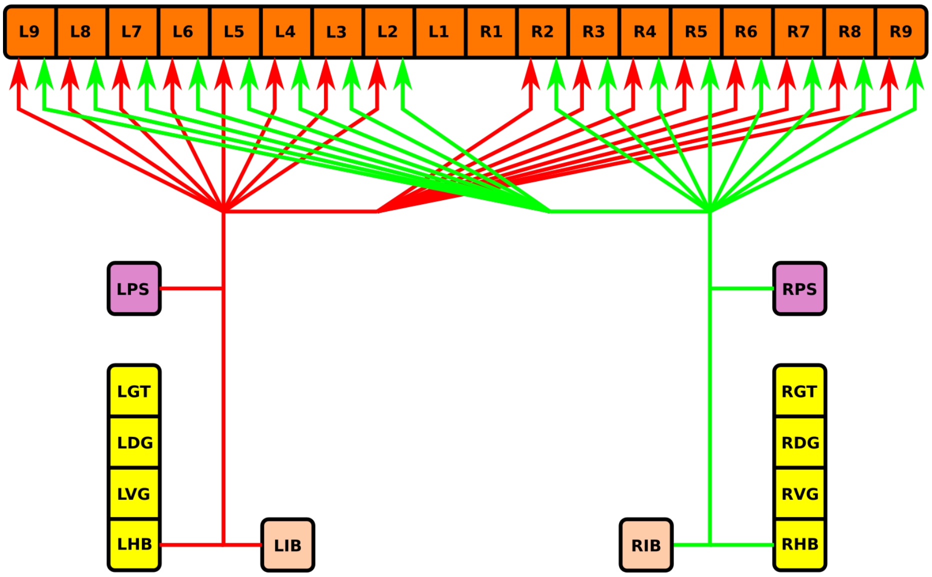

Figure S12. Neuropil innervation pattern for IB-LAL-PS-PB neurons (Tab. S6).

3.2.6 PB-EB-BU Projection Neurons

Neurons with postsynaptic arborizations in PB and presynaptic arborizations in EB and BU have been observed. These correspond to the PB-EB-LTR neurons in (Hanesch et al., 1989, Fig. 10b). Details regarding possible neuron types have not been determined.

3.2.7 PB-EB-NO Projection Neurons

Neurons with postsynaptic arborizations in PB and presynaptic arborizations in EB and NO are referred to as PEN neurons in (Lin et al., 2013, p. 1745). The constitute a cholinergic pathway (Lin et al., 2013, Fig. 7C). The connections in Tab. S7 and Fig. S13 are based upon (Wolff et al., 2015, Fig. 14a), which differ from those described in (Lin et al., 2013, Fig. 5g).

	**Label**
1	PB/L9/s-EB/6/b-no/(1,R)/b
2	PB/L8/s-EB/7/b-no/(1,R)/b
3	PB/L7/s-EB/8/b-no/(1,R)/b
4	PB/L6/s-EB/1/b-no/(1,R)/b
5	PB/L5/s-EB/2/b-no/(1,R)/b
6	PB/L4/s-EB/3/b-no/(1,R)/b
7	PB/L3/s-EB/4/b-no/(1,R)/b
8	PB/L2/s-EB/5/b-no/(1,R)/b
9	PB/R2/s-EB/5/b-NO/(1,L)/b
10	PB/R3/s-EB/6/b-NO/(1,L)/b
11	PB/R4/s-EB/7/b-NO/(1,L)/b
12	PB/R5/s-EB/8/b-NO/(1,L)/b
13	PB/R6/s-EB/1/b-NO/(1,L)/b
14	PB/R7/s-EB/2/b-NO/(1,L)/b
15	PB/R8/s-EB/3/b-NO/(1,L)/b
16	PB/R9/s-EB/4/b-NO/(1,L)/b

Table S7. PB-EB-NO neurons.

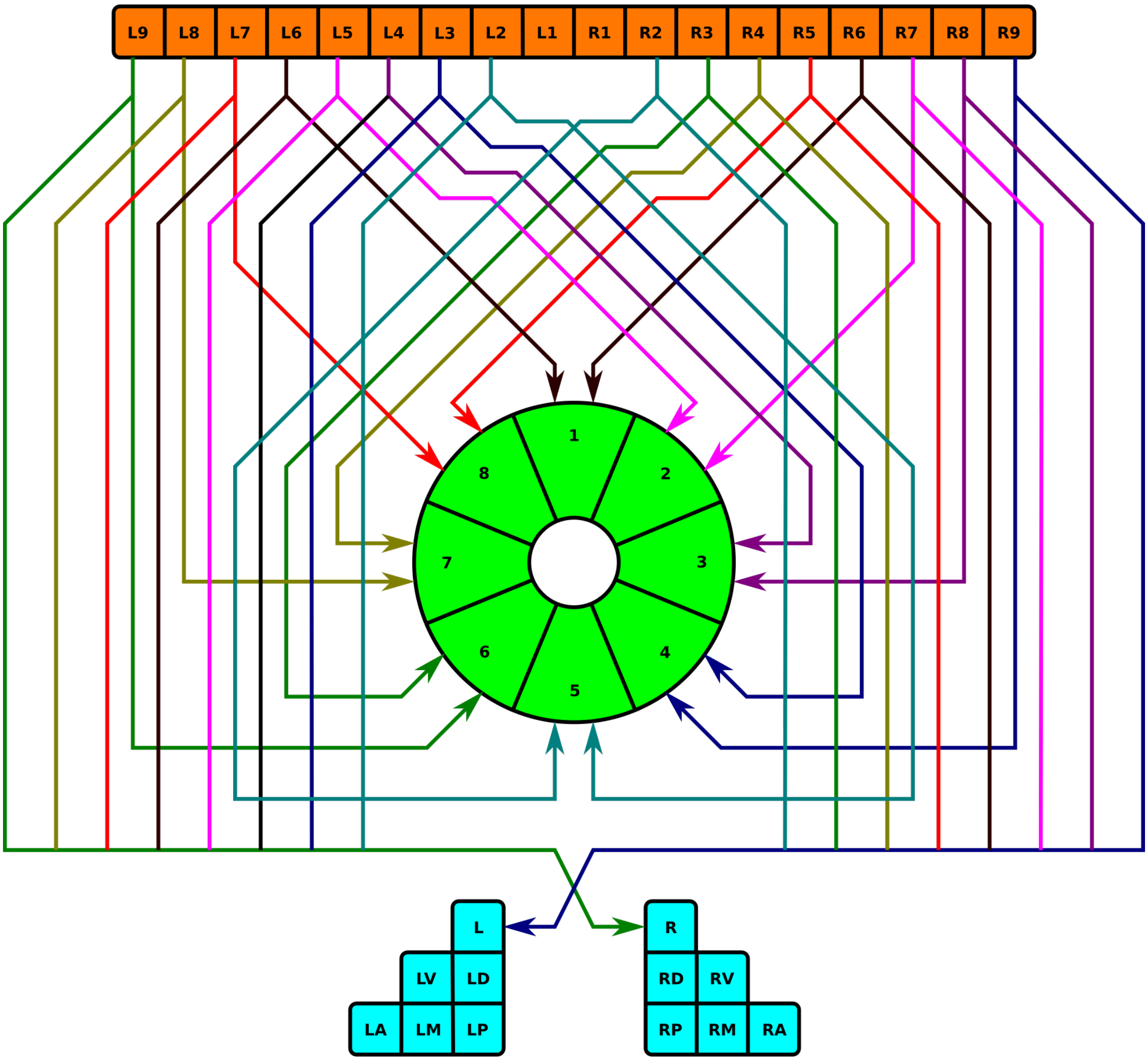

Figure S13. Neuropil innervation pattern for PB-EB-NO neurons (Tab. S7).

3.2.8 PB-EB-LAL Projection Neurons

These neurons have postsynaptic arborizations in PB and presynaptic arborizations in EB and LAL; they correspond to PB-EB-IDFP or PEI neurons in (Lin et al., 2013, Fig. 2). These neurons constitute a cholinergic pathway (Lin et al., 2013, Fig. 7C). The connections in Tab. S8 and Fig. S14 are based upon (Wolff et al., 2015, Fig. 14b), which differ from those described in (Lin et al., 2013, Fig. 5e).

	Label
1	PB/L8/s-EB/6/b-lal/RVG/b
2	PB/L7/s-EB/7/b-lal/RDG/b
3	PB/L6/s-EB/8/b-lal/RVG/b
4	PB/L5/s-EB/1/b-lal/RDG/b
5	PB/L4/s-EB/2/b-lal/RVG/b
6	PB/L3/s-EB/3/b-lal/RDG/b
7	PB/L2/s-EB/4/b-lal/RVG/b
8	PB/L1/s-EB/5/b-lal/RDG/b
9	PB/R1/s-EB/5/b-LAL/LDG/b
10	PB/R2/s-EB/4/b-LAL/LVG/b
11	PB/R3/s-EB/3/b-LAL/LDG/b
12	PB/R4/s-EB/2/b-LAL/LVG/b
13	PB/R5/s-EB/1/b-LAL/LDG/b
14	PB/R6/s-EB/8/b-LAL/LVG/b
15	PB/R7/s-EB/7/b-LAL/LDG/b
16	PB/R8/s-EB/6/b-LAL/LVG/b

Table S8. PB-EB-LAL neurons.

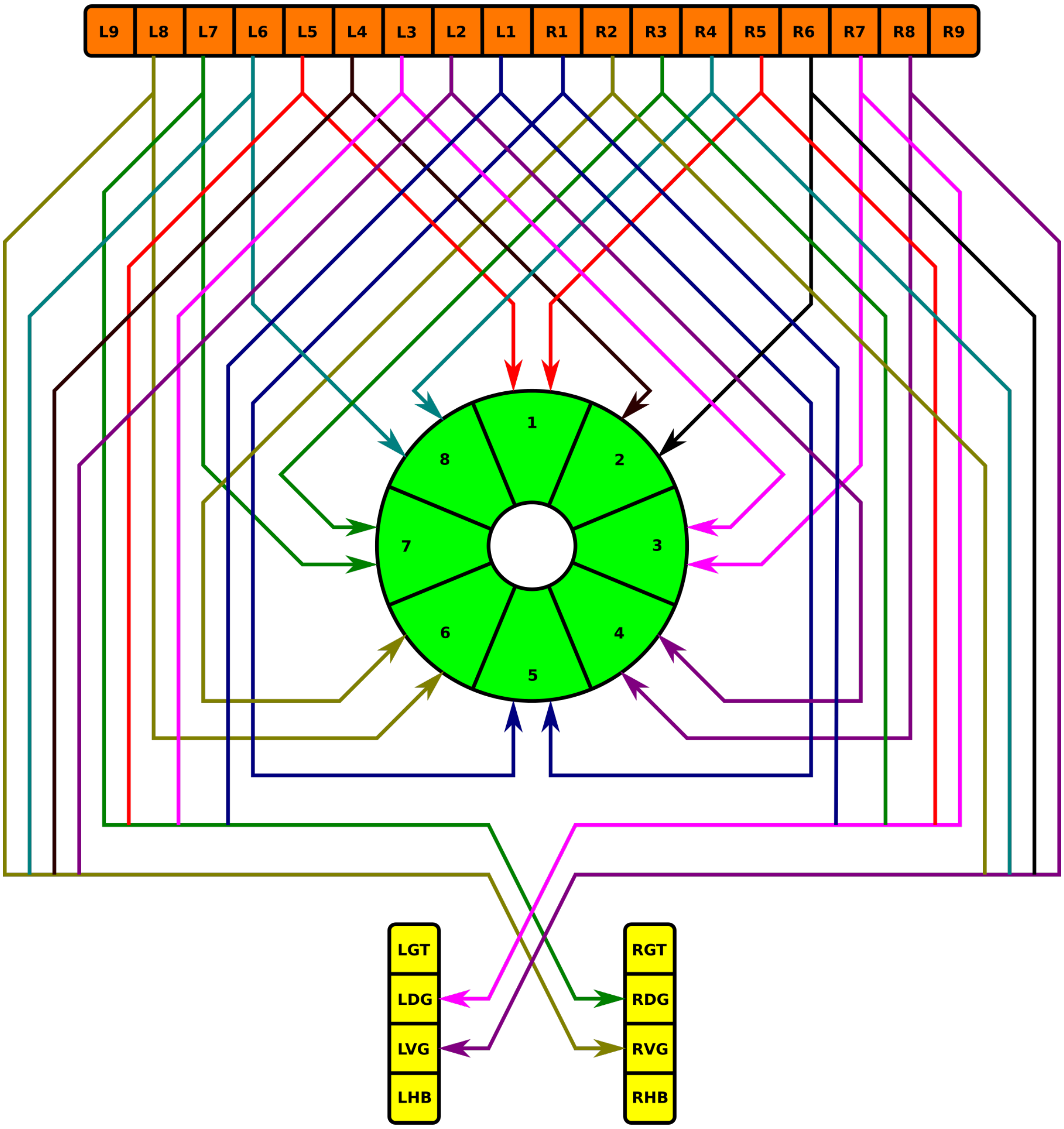

Figure S14. Neuropil innervation pattern for PB-EB-LAL neurons (Tab. S8).

3.2.9 PB-FB-CRE Projection Neurons

The neurons in Tab. S9 and Fig. S15 correspond to the PB-FB-VBO or PFI neurons that connect to RB in (Lin et al., 2013, Fig. 2). They constitute a cholinergic pathway (Lin et al., 2013, Fig. 7C).

	**Label**
1	PB/L1/s-FB/([3-4],L4)/s-CRE/LRB/b
2	PB/R1/s-FB/([3-4],L4)/s-CRE/LRB/b
3	PB/R2/s-FB/([3-4],L3)/s-CRE/LRB/b
4	PB/R3/s-FB/([3-4],L2)/s-CRE/LRB/b
5	PB/R4/s-FB/([3-4],L1)/s-CRE/LRB/b
6	PB/R4/s-FB/([3-4],R1)/s-CRE/LRB/b
7	PB/R5/s-FB/([3-4],R2)/s-CRE/LRB/b
8	PB/R6/s-FB/([3-4],R3)/s-CRE/LRB/b
9	PB/R7/s-FB/([3-4],R4)/s-CRE/LRB/b
10	PB/L7/s-FB/([3-4],L4)/s-cre/RRB/b
11	PB/L6/s-FB/([3-4],L3)/s-cre/RRB/b
12	PB/L5/s-FB/([3-4],L2)/s-cre/RRB/b
13	PB/L4/s-FB/([3-4],L1)/s-cre/RRB/b
14	PB/L4/s-FB/([3-4],R1)/s-cre/RRB/b
15	PB/L3/s-FB/([3-4],R2)/s-cre/RRB/b
16	PB/L2/s-FB/([3-4],R3)/s-cre/RRB/b
17	PB/L1/s-FB/([3-4],R4)/s-cre/RRB/b
18	PB/R1/s-FB/([3-4],R4)/s-cre/RRB/b

Table S9. PB-FB-CRE neurons.

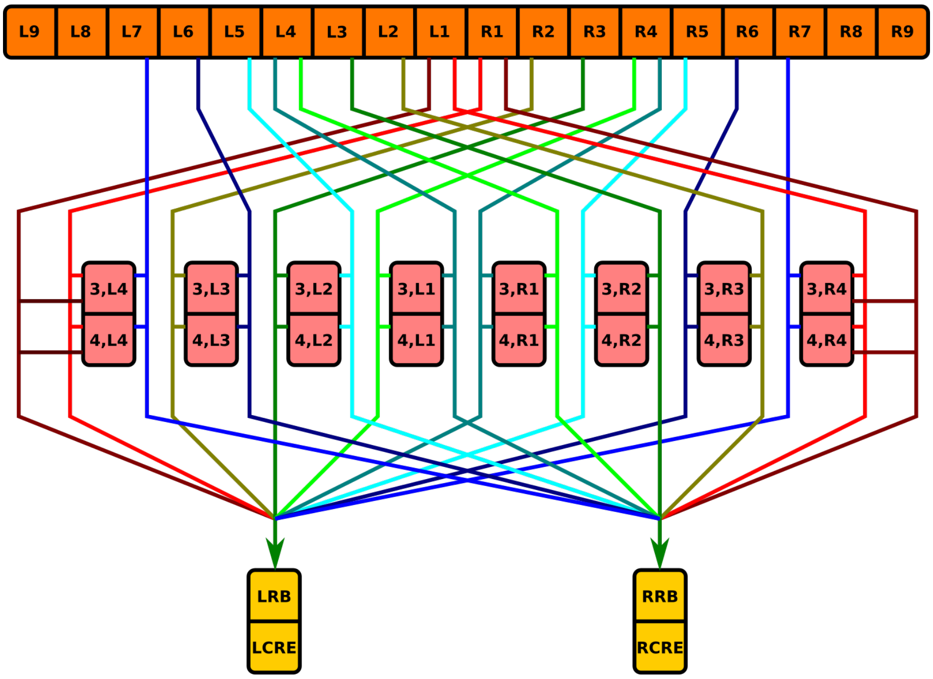

Figure S15. Neuropil innervation pattern for PB-FB-CRE neurons (Tab. S9).

3.2.10 PB-FB-NO Projection Neurons

Neurons with postsynaptic arborizations in PB and presynaptic arborizations in FB and NO are referred to as the vertical fiber system (Hanesch et al., 1989, Fig. 5b); they correspond to the PFN neurons described in (Lin et al., 2013, p. 1745). These neurons constitute a cholinergic pathway (Lin et al., 2013, Fig. 7C). The 5 sets of PB-FB-NO neurons are listed in Tabs. S10, S11, S12, S13, and S14 and depicted in Fig. S16.

	Label
1	PB/L2/s-FB/(1,R4)/b-no/(3,RP)/b
2	PB/L3/s-FB/(1,R4)/b-no/(3,RP)/b
3	PB/L4/s-FB/(1,R3)/b-no/(3,RP)/b
4	PB/L5/s-FB/(1,R2)/b-no/(3,RP)/b
5	PB/L6/s-FB/(1,R1)|(1,L1)/b-no/(3,RP)/b
6	PB/L7/s-FB/(1,L2)/b-no/(3,RP)/b
7	PB/L8/s-FB/(1,L3)/b-no/(3,RP)/b
8	PB/L9/s-FB/(1,L4)/b-no/(3,RP)/b
9	PB/R2/s-FB/(1,L4)/b-NO/(3,LP)/b
10	PB/R3/s-FB/(1,L4)/b-NO/(3,LP)/b
11	PB/R4/s-FB/(1,L3)/b-NO/(3,LP)/b
12	PB/R5/s-FB/(1,L2)/b-NO/(3,LP)/b
13	PB/R6/s-FB/(1,L1)|(1,R1)/b-NO/(3,LP)/b
14	PB/R7/s-FB/(1,R2)/b-NO/(3,LP)/b
15	PB/R8/s-FB/(1,R3)/b-NO/(3,LP)/b
16	PB/R9/s-FB/(1,R4)/b-NO/(3,LP)/b

Table S10. PB-FB-NO neurons innervating region (3,P) of NO.

	Label
1	PB/L2/s-FB/(1,R4)/b-no/(3,RM)/b
2	PB/L3/s-FB/(1,R4)/b-no/(3,RM)/b
3	PB/L4/s-FB/(1,R3)/b-no/(3,RM)/b
4	PB/L5/s-FB/(1,R2)/b-no/(3,RM)/b
5	PB/L6/s-FB/(1,R1)|(1,L1)/b-no/(3,RM)/b
6	PB/L7/s-FB/(1,L2)/b-no/(3,RM)/b
7	PB/L8/s-FB/(1,L3)/b-no/(3,RM)/b
8	PB/L9/s-FB/(1,L4)/b-no/(3,RM)/b
9	PB/R2/s-FB/(1,L4)/b-NO/(3,LM)/b
10	PB/R3/s-FB/(1,L4)/b-NO/(3,LM)/b
11	PB/R4/s-FB/(1,L3)/b-NO/(3,LM)/b
12	PB/R5/s-FB/(1,L2)/b-NO/(3,LM)/b
13	PB/R6/s-FB/(1,L1)|(1,R1)/b-NO/(3,LM)/b
14	PB/R7/s-FB/(1,R2)/b-NO/(3,LM)/b
15	PB/R8/s-FB/(1,R3)/b-NO/(3,LM)/b
16	PB/R9/s-FB/(1,R4)/b-NO/(3,LM)/b

Table S11. PB-FB-NO neurons innervating region (3,M) of NO.

	Label
1	PB/L2/s-FB/(2,R4)/b-no/(3,RA)/b
2	PB/L3/s-FB/(2,R4)/b-no/(3,RA)/b
3	PB/L4/s-FB/(2,R3)/b-no/(3,RA)/b
4	PB/L5/s-FB/(2,R2)/b-no/(3,RA)/b
5	PB/L6/s-FB/(2,R1)|(1,L1)/b-no/(3,RA)/b
6	PB/L7/s-FB/(2,L2)/b-no/(3,RA)/b
7	PB/L8/s-FB/(2,L3)/b-no/(3,RA)/b
8	PB/L9/s-FB/(2,L4)/b-no/(3,RA)/b
9	PB/R2/s-FB/(2,L4)/b-NO/(3,LA)/b
10	PB/R3/s-FB/(2,L4)/b-NO/(3,LA)/b
11	PB/R4/s-FB/(2,L3)/b-NO/(3,LA)/b
12	PB/R5/s-FB/(2,L2)/b-NO/(3,LA)/b
13	PB/R6/s-FB/(2,L1)|(1,R1)/b-NO/(3,LA)/b
14	PB/R7/s-FB/(2,R2)/b-NO/(3,LA)/b
15	PB/R8/s-FB/(2,R3)/b-NO/(3,LA)/b
16	PB/R9/s-FB/(2,R4)/b-NO/(3,LA)/b

Table S12. PB-FB-NO neurons innervating region (3,A) of NO.

	Label
1	PB/L2/s-FB/(3,R4)/b-no/(2,RD)/b
2	PB/L3/s-FB/(3,R4)/b-no/(2,RD)/b
3	PB/L4/s-FB/(3,R3)/b-no/(2,RD)/b
4	PB/L5/s-FB/(3,R2)/b-no/(2,RD)/b
5	PB/L6/s-FB/(3,R1)|(1,L1)/b-no/(2,RD)/b
6	PB/L7/s-FB/(3,L2)/b-no/(2,RD)/b
7	PB/L8/s-FB/(3,L3)/b-no/(2,RD)/b
8	PB/L9/s-FB/(3,L4)/b-no/(2,RD)/b
9	PB/R2/s-FB/(3,L4)/b-NO/(2,LD)/b
10	PB/R3/s-FB/(3,L4)/b-NO/(2,LD)/b
11	PB/R4/s-FB/(3,L3)/b-NO/(2,LD)/b
12	PB/R5/s-FB/(3,L2)/b-NO/(2,LD)/b
13	PB/R6/s-FB/(3,L1)|(1,R1)/b-NO/(2,LD)/b
14	PB/R7/s-FB/(3,R2)/b-NO/(2,LD)/b
15	PB/R8/s-FB/(3,R3)/b-NO/(2,LD)/b
16	PB/R9/s-FB/(3,R4)/b-NO/(2,LD)/b

Table S13. PB-FB-NO neurons innervating region (2,D) of NO.

	Label
1	PB/L2/s-FB/(3,R4)/b-no/(2,RV)/b
2	PB/L3/s-FB/(3,R4)/b-no/(2,RV)/b
3	PB/L4/s-FB/(3,R3)/b-no/(2,RV)/b
4	PB/L5/s-FB/(3,R2)/b-no/(2,RV)/b
5	PB/L6/s-FB/(3,R1)|(1,L1)/b-no/(2,RV)/b
6	PB/L7/s-FB/(3,L2)/b-no/(2,RV)/b
7	PB/L8/s-FB/(3,L3)/b-no/(2,RV)/b
8	PB/L9/s-FB/(3,L4)/b-no/(2,RV)/b
9	PB/R2/s-FB/(3,L4)/b-NO/(2,LV)/b
10	PB/R3/s-FB/(3,L4)/b-NO/(2,LV)/b
11	PB/R4/s-FB/(3,L3)/b-NO/(2,LV)/b
12	PB/R5/s-FB/(3,L2)/b-NO/(2,LV)/b
13	PB/R6/s-FB/(3,L1)|(1,R1)/b-NO/(2,LV)/b
14	PB/R7/s-FB/(3,R2)/b-NO/(2,LV)/b
15	PB/R8/s-FB/(3,R3)/b-NO/(2,LV)/b
16	PB/R9/s-FB/(3,R4)/b-NO/(2,LV)/b

Table S14. PB-FB-NO neurons innervating region (2,V) of NO.

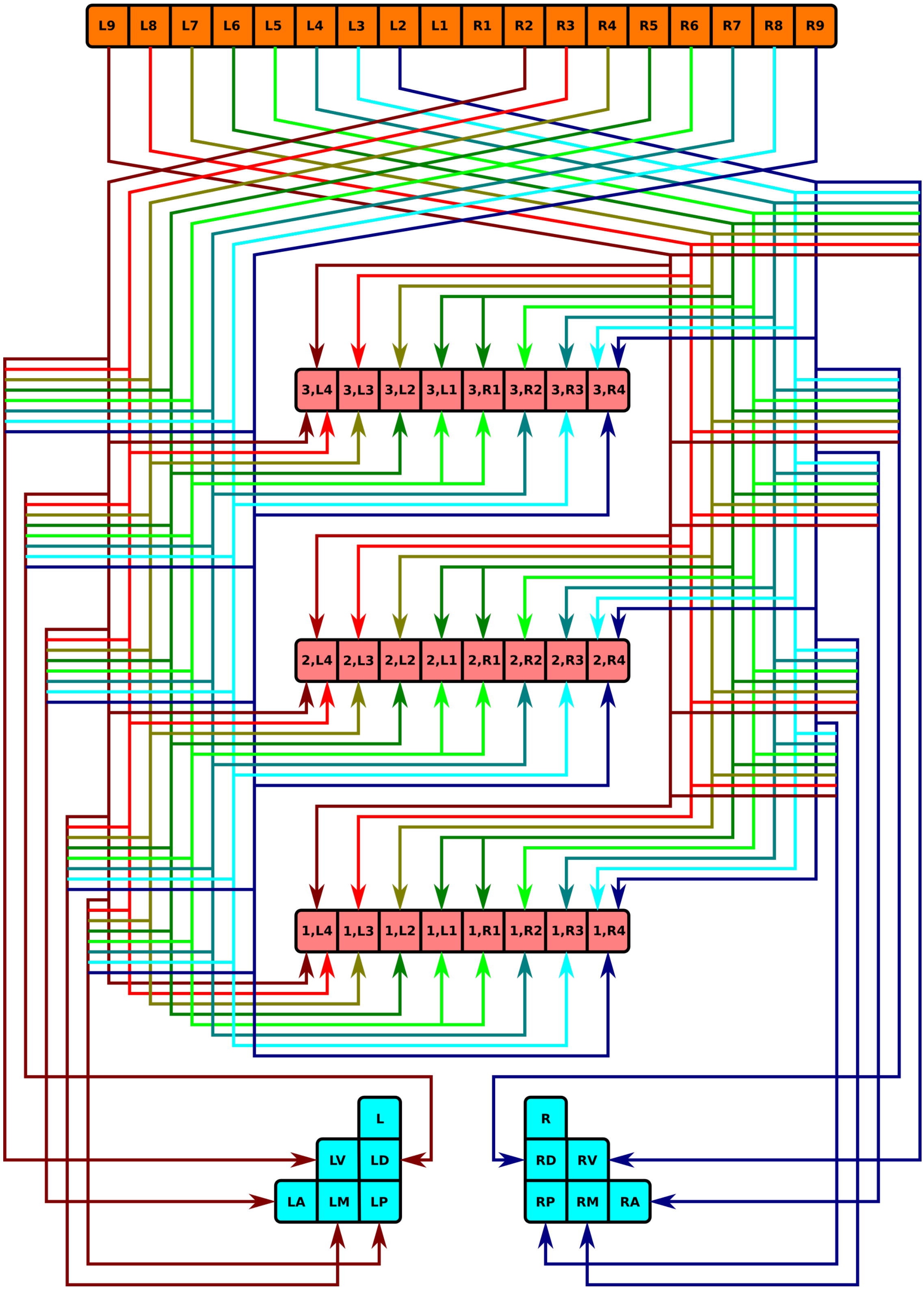

Figure S16. Neuropil innervation pattern for PB-FB-NO neurons (Tabs. S10, S11, S12, S13, S14).

3.2.11 PB-FB-LAL Projection Neurons

Neurons with postsynaptic arborizations in PB and FB and presynaptic arborizations in LAL are referred to as PFI neurons in (Lin et al., 2013, p. 1745). They correspond to the PB-FB-VBO or PFI neurons that connect to HB in (Lin et al., 2013, Fig. 2), and are also referred to as the horizontal fiber system (Hanesch et al., 1989, Fig. 6b). [1] These neurons constitute a cholinergic pathway (Lin et al., 2013, Fig. 7C).

<table>
<tr><th></th><th>Label</th></tr>
<tr><td>1</td><td>PB/L1/s-FB/(2,L[3-4])/s-LAL/LHB/b</td></tr>
<tr><td>2</td><td>PB/R1|L1/s-FB/(2,L[3-4])/s-LAL/LHB/b</td></tr>
<tr><td>3</td><td>PB/R1/s-FB/(2,L[2-3])/s-LAL/LHB/b</td></tr>
<tr><td>4</td><td>PB/R2/s-FB/(2,L[1-2])/s-LAL/LHB/b</td></tr>
<tr><td>5</td><td>PB/R3/s-FB/(2,[L1,R1])/s-LAL/LHB/b</td></tr>
<tr><td>6</td><td>PB/R4/s-FB/(2,R[1-2])/s-LAL/LHB/b</td></tr>
<tr><td>7</td><td>PB/R5/s-FB/(2,R[2-3])/s-LAL/LHB/b</td></tr>
<tr><td>8</td><td>PB/R6/s-FB/(2,R[3-4])/s-LAL/LHB/b</td></tr>
<tr><td>9</td><td>PB/R7/s-FB/(2,R[3-4])/s-LAL/LHB/b</td></tr>
<tr><td>10</td><td>PB/L7/s-FB/(2,L[3-4])/s-lal/RHB/b</td></tr>
<tr><td>11</td><td>PB/L6/s-FB/(2,L[3-4])/s-lal/RHB/b</td></tr>
<tr><td>12</td><td>PB/L5/s-FB/(2,L[2-3])/s-lal/RHB/b</td></tr>
<tr><td>13</td><td>PB/L4/s-FB/(2,L[1-2])/s-lal/RHB/b</td></tr>
<tr><td>14</td><td>PB/L3/s-FB/(2,[R1,L1])/s-lal/RHB/b</td></tr>
<tr><td>15</td><td>PB/L2/s-FB/(2,R[1-2])/s-lal/RHB/b</td></tr>
<tr><td>16</td><td>PB/L1/s-FB/(2,R[2-3])/s-lal/RHB/b</td></tr>
<tr><td>17</td><td>PB/L1|R1/s-FB/(2,R[3-4])/s-lal/RHB/b</td></tr>
<tr><td>18</td><td>PB/R1/s-FB/(2,R[3-4])/s-lal/RHB/b</td></tr>
</table>

Table S15. PB-FB-LAL neurons innervating layer 2 of FB (Lin et al., 2013, Fig. 6F).

[1] These sources appear to consider CRE as part of LAL; this document treats CRE separately.

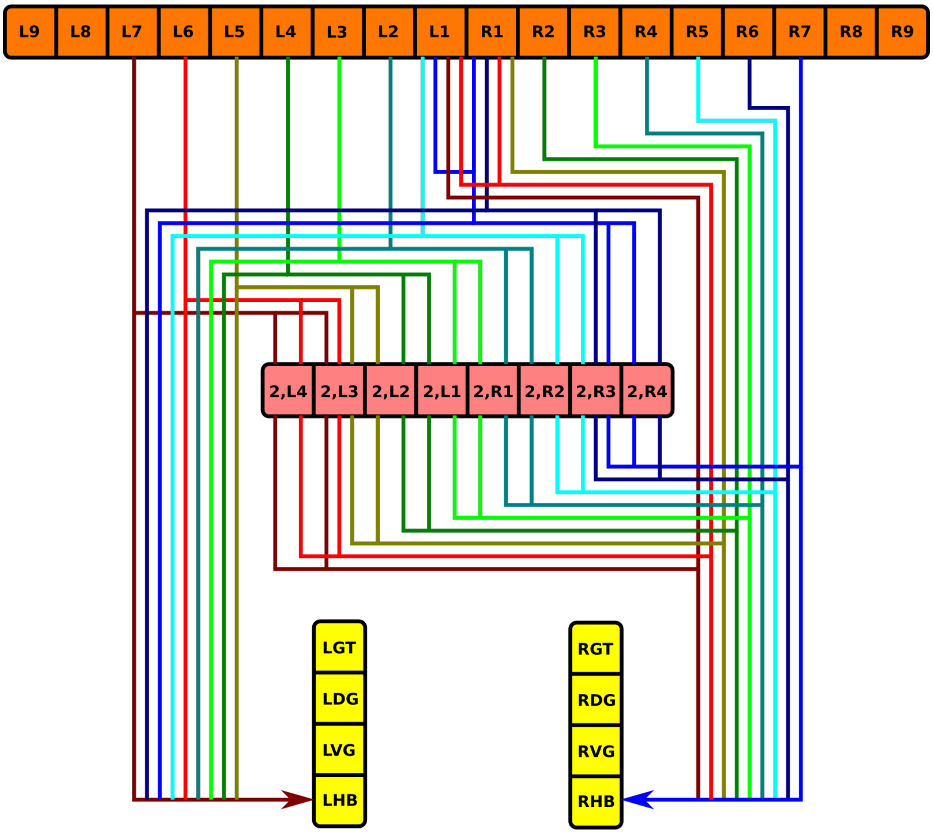

Figure S17. Neuropil innervation pattern for PB-FB-LAL neurons innervating layer 2 of FB (Tab. S15).

	Label
1	PB/L1/s-FB/([1-4],L[3-4])/s-LAL/LHB/b
2	PB/L1|R1/s-FB/([1-4],L[2-3])/s-LAL/LHB/b
3	PB/R1/s-FB/([1-4],L[1-2])/s-LAL/LHB/b
4	PB/R2/s-FB/([1-4],[L1,R1])/s-LAL/LHB/b
5	PB/R3/s-FB/([1-4],R[1-2])/s-LAL/LHB/b
6	PB/R4/s-FB/([1-4],R[2-3])/s-LAL/LHB/b
7	PB/R5/s-FB/([1-4],R[3-4])/s-LAL/LHB/b
8	PB/R6/s-FB/([1-4],R[3-4])/s-LAL/LHB/b
9	PB/L6/s-FB/([1-4],L[3-4])/s-lal/RHB/b
10	PB/L5/s-FB/([1-4],L[3-4])/s-lal/RHB/b
11	PB/L4/s-FB/([1-4],L[2-3])/s-lal/RHB/b
12	PB/L3/s-FB/([1-4],L[1-2])/s-lal/RHB/b
13	PB/L2/s-FB/([1-4],[L1,R1])/s-lal/RHB/b
14	PB/L1/s-FB/([1-4],R[1-2])/s-lal/RHB/b
15	PB/L1|R1/s-FB/([1-4],R[2-3])/s-lal/RHB/b
16	PB/R1/s-FB/([1-4],R[3-4])/s-lal/RHB/b

Table S16. PB-FB-LAL neurons (Lin et al., 2013, Fig. 6G).

	Label
1	PB/L3/s-FB/([1-4],L[3-4])/s-LAL/LHB/b-lal/RHB/b
2	PB/L1/s-FB/([1-4],L[1-2])/s-LAL/LHB/b-lal/RHB/b
3	PB/R1/s-FB/([1-4],R[1-2])/s-LAL/LHB/b-lal/RHB/b
4	PB/R3/s-FB/([1-4],R[3-4])/s-LAL/LHB/b-lal/RHB/b

Table S17. PB-FB-LAL neurons (Lin et al., 2013, Fig. 6G).

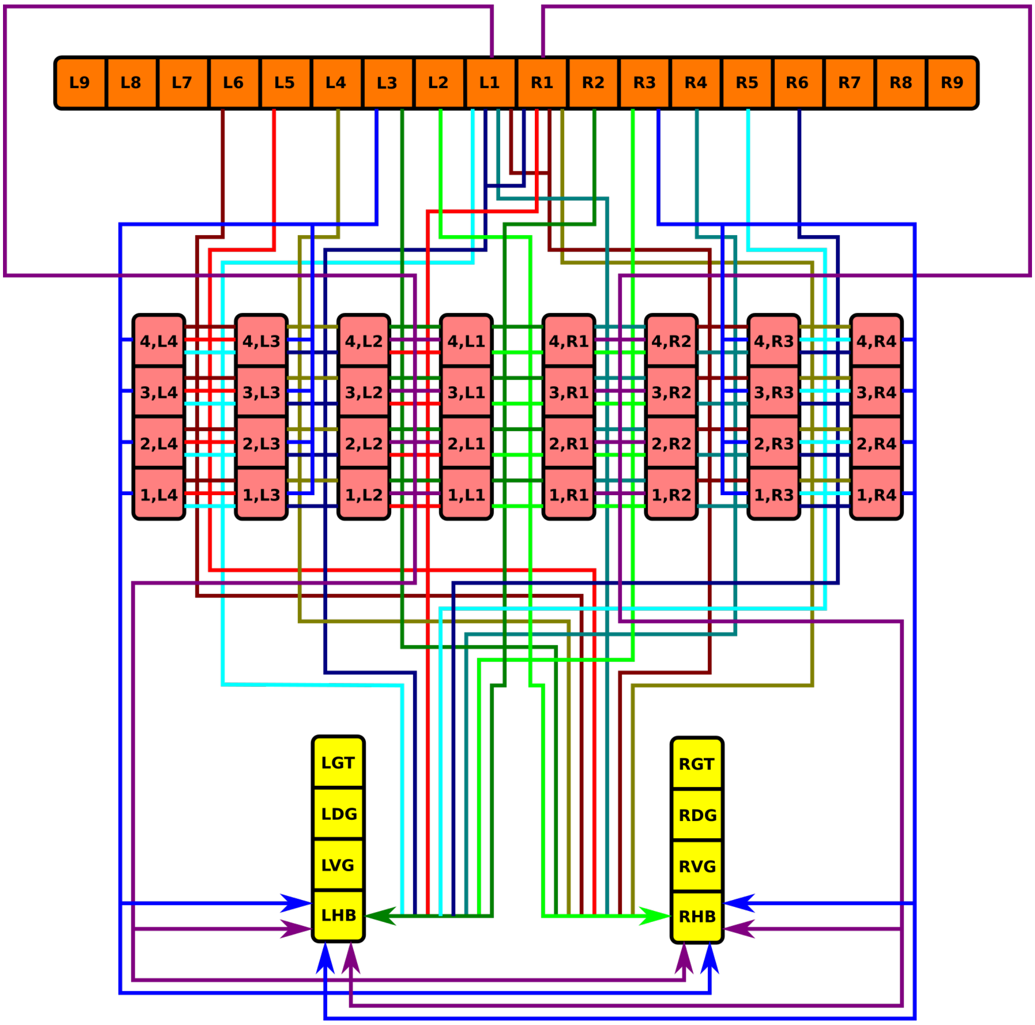

Figure S18. Neuropil innervation pattern for PB-FB-LAL neurons (Tab. S16, S17).

3.2.12 WED-PS-PB Projection Neurons

The neurons in Tab. S18 correspond to CCP-VMP-PB or CVP neurons in (Lin et al., 2013, Fig. 2) and receive input from the vision system. They constitute a cholinergic pathway (Lin et al., 2013, Fig. 7C).

<table>
<tr><th></th><th>Label</th></tr>
<tr><td>1</td><td>WED/L/s-PS/L/s-PB/L2|L3/b</td></tr>
<tr><td>2</td><td>WED/L/s-PS/L/s-PB/L3|L4/b</td></tr>
<tr><td>3</td><td>WED/L/s-PS/L/s-PB/L4|L5/b</td></tr>
<tr><td>4</td><td>WED/L/s-PS/L/s-PB/L5|L6/b</td></tr>
<tr><td>5</td><td>WED/L/s-PS/L/s-PB/L6|L7/b</td></tr>
<tr><td>6</td><td>WED/L/s-PS/L/s-PB/L7|L8/b</td></tr>
<tr><td>7</td><td>WED/L/s-PS/L/s-PB/L8|L9/b</td></tr>
<tr><td>8</td><td>wed/R/s-ps/R/s-PB/L2|L3/b</td></tr>
<tr><td>9</td><td>wed/R/s-ps/R/s-PB/L3|L4/b</td></tr>
<tr><td>10</td><td>wed/R/s-ps/R/s-PB/L4|L5/b</td></tr>
<tr><td>11</td><td>wed/R/s-ps/R/s-PB/L5|L6/b</td></tr>
<tr><td>12</td><td>wed/R/s-ps/R/s-PB/L6|L7/b</td></tr>
<tr><td>13</td><td>wed/R/s-ps/R/s-PB/L7|L8/b</td></tr>
<tr><td>14</td><td>wed/R/s-ps/R/s-PB/L8|L9/b</td></tr>
<tr><td>15</td><td>WED/L/s-PS/L/s-PB/R2|R3/b</td></tr>
<tr><td>16</td><td>WED/L/s-PS/L/s-PB/R3|R4/b</td></tr>
<tr><td>17</td><td>WED/L/s-PS/L/s-PB/R4|R5/b</td></tr>
<tr><td>18</td><td>WED/L/s-PS/L/s-PB/R5|R6/b</td></tr>
<tr><td>19</td><td>WED/L/s-PS/L/s-PB/R6|R7/b</td></tr>
<tr><td>20</td><td>WED/L/s-PS/L/s-PB/R7|R8/b</td></tr>
<tr><td>21</td><td>WED/L/s-PS/L/s-PB/R8|R9/b</td></tr>
<tr><td>22</td><td>wed/R/s-ps/R/s-PB/R2|R3/b</td></tr>
<tr><td>23</td><td>wed/R/s-ps/R/s-PB/R3|R4/b</td></tr>
<tr><td>24</td><td>wed/R/s-ps/R/s-PB/R4|R5/b</td></tr>
<tr><td>25</td><td>wed/R/s-ps/R/s-PB/R5|R6/b</td></tr>
<tr><td>26</td><td>wed/R/s-ps/R/s-PB/R6|R7/b</td></tr>
<tr><td>27</td><td>wed/R/s-ps/R/s-PB/R7|R8/b</td></tr>
<tr><td>28</td><td>wed/R/s-ps/R/s-PB/R8|R9/b</td></tr>
</table>

Table S18. WED-PS-PB neurons.

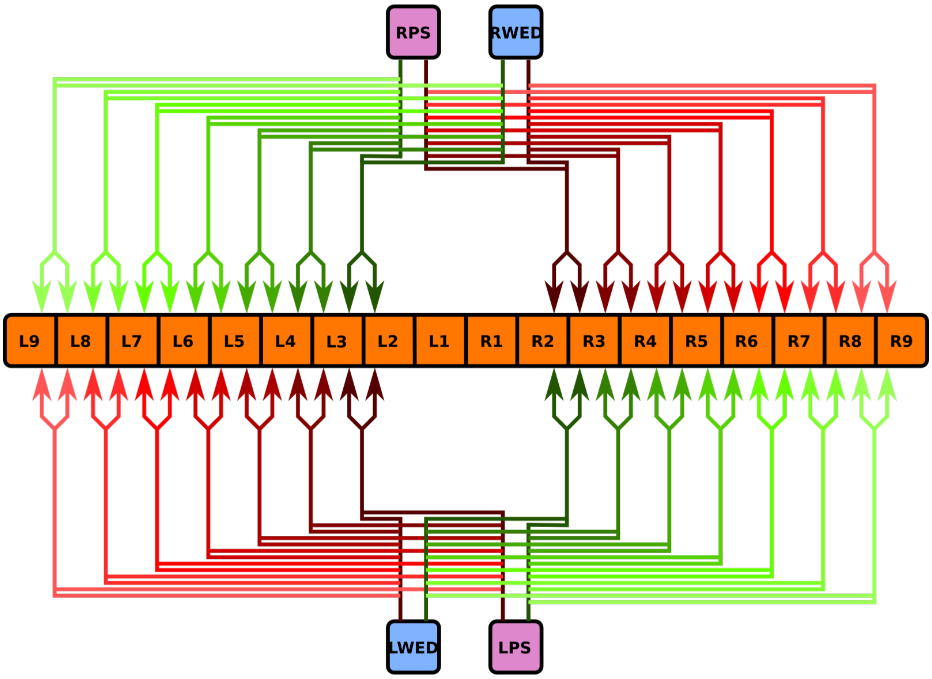

Figure S19. Neuropil innervation pattern for WED-PS-PB neurons (Tab. S18).

4 HYPOTHESIZED NEURONS IN THE CENTRAL COMPLEX

The arborization patterns of the following neuron families is inferred from currently available data; however, they have not been systematically characterized.

4.1 BU-EB Neurons

Ring Neuron Name	Microglomerulus Range	Label
R1	1..16	BU/Lx/s-EB/(LR[1-8],[M,A],1)/b
		bu/Rx/s-EB/(LR[1-8],[M,A],1)/b
R2	33..48	BU/Lx/s-EB/(LR[1-8],A,[3,4])/b
		bu/Rx/s-EB/(LR[1-8],A,[3,4])/b
R3	17..32	BU/Lx/s-EB/(LR[1-8],A,[1,2])/b
		bu/Rx/s-EB/(LR[1-8],A,[1,2])/b
R4m	49..64	BU/Lx/s-EB/(LR[1-8],A,4)/b
		bu/Rx/s-EB/(LR[1-8],A,4)/b
R4d	65..80	BU/Lx/s-EB/(LR[1-8],M,4)/b
		bu/Rx/s-EB/(LR[1-8],M,4)/b

Table S19. Hypothesized arborizations of BU-EB neurons. Each microglomerulus corresponds to a single neuron, where the “x” character in the neuron label is replaced with the microglomerulus number.

4.2 FB Local Neurons

Based upon the pontine neuron family described in (Hanesch et al., 1989, p. 349), additional local FB neurons were hypothesized as connecting

- nonadjacent segments in layers 1, 2, 4, and 5 (Tab. S20);
- adjacent segments within the same layer in layers 1-5 (Hanesch et al., 1989, Fig. 9a) (Tab. S21).
- adjacent layers within the same segment for layers 1-5 (Hanesch et al., 1989, Fig. 9a) (Tab. S22).
- nonadjacent layers within the same segment, with both presynaptic and postsynaptic terminals in each layer (Hanesch et al., 1989, Fig. 9b) (Tab. S23); based upon the latter source, we assume two sets of neurons connecting layers 1 and 8 and 2 and 7, respectively.

	Label
1	FB/(1,L4)/s-FB/(1,R1)/b
2	FB/(1,L3)/s-FB/(1,R2)/b
3	FB/(1,L2)/s-FB/(1,R3)/b
4	FB/(1,L1)/s-FB/(1,R4)/b
5	FB/(1,R4)/s-FB/(1,L1)/b
6	FB/(1,R3)/s-FB/(1,L2)/b
7	FB/(1,R2)/s-FB/(1,L3)/b
8	FB/(1,R1)/s-FB/(1,L4)/b
9	FB/(2,L4)/s-FB/(2,R1)/b
10	FB/(2,L3)/s-FB/(2,R2)/b
11	FB/(2,L2)/s-FB/(2,R3)/b
12	FB/(2,L1)/s-FB/(2,R4)/b
13	FB/(2,R4)/s-FB/(2,L1)/b
14	FB/(2,R3)/s-FB/(2,L2)/b
15	FB/(2,R2)/s-FB/(2,L3)/b
16	FB/(2,R1)/s-FB/(2,L4)/b
17	FB/(4,L4)/s-FB/(4,R1)/b
18	FB/(4,L3)/s-FB/(4,R2)/b
19	FB/(4,L2)/s-FB/(4,R3)/b
20	FB/(4,L1)/s-FB/(4,R4)/b
21	FB/(4,R4)/s-FB/(4,L1)/b
22	FB/(4,R3)/s-FB/(4,L2)/b
23	FB/(4,R2)/s-FB/(4,L3)/b
24	FB/(4,R1)/s-FB/(4,L4)/b
25	FB/(5,L4)/s-FB/(5,R1)/b
26	FB/(5,L3)/s-FB/(5,R2)/b
27	FB/(5,L2)/s-FB/(5,R3)/b
28	FB/(5,L1)/s-FB/(5,R4)/b
29	FB/(5,R4)/s-FB/(5,L1)/b
30	FB/(5,R3)/s-FB/(5,L2)/b
31	FB/(5,R2)/s-FB/(5,L3)/b
32	FB/(5,R1)/s-FB/(5,L4)/b

Table S20. Hypothesized FB local neurons linking segments in layers 1, 2, 4, and 5, respectively.

	**Label**
1	FB/(1,L4)/s-FB/(1,L3)/b
2	FB/(1,L3)/s-FB/(1,L2)/b
3	FB/(1,L2)/s-FB/(1,L1)/b
4	FB/(1,L1)/s-FB/(1,R1)/b
5	FB/(1,R1)/s-FB/(1,R2)/b
6	FB/(1,R2)/s-FB/(1,R3)/b
7	FB/(1,R3)/s-FB/(1,R4)/b
8	FB/(2,L4)/s-FB/(2,L3)/b
9	FB/(2,L3)/s-FB/(2,L2)/b
10	FB/(2,L2)/s-FB/(2,L1)/b
11	FB/(2,L1)/s-FB/(2,R1)/b
12	FB/(2,R1)/s-FB/(2,R2)/b
13	FB/(2,R2)/s-FB/(2,R3)/b
14	FB/(2,R3)/s-FB/(2,R4)/b
15	FB/(3,L4)/s-FB/(3,L3)/b
16	FB/(3,L3)/s-FB/(3,L2)/b
17	FB/(3,L2)/s-FB/(3,L1)/b
18	FB/(3,L1)/s-FB/(3,R1)/b
19	FB/(3,R1)/s-FB/(3,R2)/b
20	FB/(3,R2)/s-FB/(3,R3)/b
21	FB/(3,R3)/s-FB/(3,R4)/b
22	FB/(4,L4)/s-FB/(4,L3)/b
23	FB/(4,L3)/s-FB/(4,L2)/b
24	FB/(4,L2)/s-FB/(4,L1)/b
25	FB/(4,L1)/s-FB/(4,R1)/b
26	FB/(4,R1)/s-FB/(4,R2)/b
27	FB/(4,R2)/s-FB/(4,R3)/b
28	FB/(4,R3)/s-FB/(4,R4)/b
29	FB/(5,L4)/s-FB/(5,L3)/b
30	FB/(5,L3)/s-FB/(5,L2)/b
31	FB/(5,L2)/s-FB/(5,L1)/b
32	FB/(5,L1)/s-FB/(5,R1)/b
33	FB/(5,R1)/s-FB/(5,R2)/b
34	FB/(5,R2)/s-FB/(5,R3)/b
35	FB/(5,R3)/s-FB/(5,R4)/b

Table S21. Hypothesized FB local neurons linking adjacent segments within the same layer in layers 1-5.

	Label
1	FB/(5,L1)/s-FB/(4,L1)/b
2	FB/(4,L1)/s-FB/(3,L1)/b
3	FB/(3,L1)/s-FB/(2,L1)/b
4	FB/(2,L1)/s-FB/(1,L1)/b
5	FB/(5,L2)/s-FB/(4,L2)/b
6	FB/(4,L2)/s-FB/(3,L2)/b
7	FB/(3,L2)/s-FB/(2,L2)/b
8	FB/(2,L2)/s-FB/(1,L2)/b
9	FB/(5,L3)/s-FB/(4,L3)/b
10	FB/(4,L3)/s-FB/(3,L3)/b
11	FB/(3,L3)/s-FB/(2,L3)/b
12	FB/(2,L3)/s-FB/(1,L3)/b
13	FB/(5,L4)/s-FB/(4,L4)/b
14	FB/(4,L4)/s-FB/(3,L4)/b
15	FB/(3,L4)/s-FB/(2,L4)/b
16	FB/(2,L4)/s-FB/(1,L4)/b
17	FB/(5,R1)/s-FB/(4,R1)/b
18	FB/(4,R1)/s-FB/(3,R1)/b
19	FB/(3,R1)/s-FB/(2,R1)/b
20	FB/(2,R1)/s-FB/(1,R1)/b
21	FB/(5,R2)/s-FB/(4,R2)/b
22	FB/(4,R2)/s-FB/(3,R2)/b
23	FB/(3,R2)/s-FB/(2,R2)/b
24	FB/(2,R2)/s-FB/(1,R2)/b
25	FB/(5,R3)/s-FB/(4,R3)/b
26	FB/(4,R3)/s-FB/(3,R3)/b
27	FB/(3,R3)/s-FB/(2,R3)/b
28	FB/(2,R3)/s-FB/(1,R3)/b
29	FB/(5,R4)/s-FB/(4,R4)/b
30	FB/(4,R4)/s-FB/(3,R4)/b
31	FB/(3,R4)/s-FB/(2,R4)/b
32	FB/(2,R4)/s-FB/(1,R4)/b

Table S22. Hypothesized FB local neurons linking adjacent layers within the same segment for layers 1-5.

	**Label**
1	FB/(1,L1)/sb-FB/(8,L1)/sb
2	FB/(1,L2)/sb-FB/(8,L2)/sb
3	FB/(1,L3)/sb-FB/(8,L3)/sb
4	FB/(1,L4)/sb-FB/(8,L4)/sb
5	FB/(1,R1)/sb-FB/(8,R1)/sb
6	FB/(1,R2)/sb-FB/(8,R2)/sb
7	FB/(1,R3)/sb-FB/(8,R3)/sb
8	FB/(1,R4)/sb-FB/(8,R4)/sb
9	FB/(2,L1)/sb-FB/(7,L1)/sb
10	FB/(2,L2)/sb-FB/(7,L2)/sb
11	FB/(2,L3)/sb-FB/(7,L3)/sb
12	FB/(2,L4)/sb-FB/(7,L4)/sb
13	FB/(2,R1)/sb-FB/(7,R1)/sb
14	FB/(2,R2)/sb-FB/(7,R2)/sb
15	FB/(2,R3)/sb-FB/(7,R3)/sb
16	FB/(2,R4)/sb-FB/(7,R4)/sb

Table S23. Hypothesized FB local neurons linking nonadjacent layers within the same segment.

4.3 PB Local Neurons in *No Bridge* Mutant

<table>
<tr><th></th><th>Label</th></tr>
<tr><td>1</td><td>PB/R9/b-PB/R[4-8]/s</td></tr>
<tr><td>2</td><td>PB/L9/b-PB/L[4-8]/s</td></tr>
<tr><td>3</td><td>PB/L2/b-PB/L[1-9]/s</td></tr>
<tr><td>4</td><td>PB/R7/b-PB/R[1-9]/s</td></tr>
<tr><td>5</td><td>PB/L7/b-PB/L[1-9]/s</td></tr>
<tr><td>6</td><td>PB/R2/b-PB/R[1-9]/s</td></tr>
<tr><td>7</td><td>PB/L3/b-PB/L[1-9]/s</td></tr>
<tr><td>8</td><td>PB/R6/b-PB/R[1-9]/s</td></tr>
<tr><td>9</td><td>PB/L6/b-PB/L[1-9]/s</td></tr>
<tr><td>10</td><td>PB/R3/b-PB/R[1-9]/s</td></tr>
<tr><td>11</td><td>PB/L4/b-PB/L[1-9]/s</td></tr>
<tr><td>12</td><td>PB/R5/b-PB/R[1-9]/s</td></tr>
<tr><td>13</td><td>PB/L5/b-PB/L[1-9]/s</td></tr>
<tr><td>14</td><td>PB/R4/b-PB/R[1-9]/s</td></tr>
<tr><td>15</td><td>PB/R8/b-PB/R[1-9]/s</td></tr>
<tr><td>16</td><td>PB/L1|L9/b-PB/L[1-9]/s</td></tr>
<tr><td>17</td><td>PB/L8/b-PB/L[1-9]/s</td></tr>
<tr><td>18</td><td>PB/R1|R9/b-PB/R[1-9]/s</td></tr>
</table>

Table S24. Hypothesized PB local neurons in the *no bridge* mutant.

REFERENCES

Chiang, A.-S., Lin, C.-Y., Chuang, C.-C., Chang, H.-M., Hsieh, C.-H., Yeh, C.-W., et al. (2011). Three-dimensional reconstruction of brain-wide wiring networks in Drosophila at single-cell resolution. Current Biology 21, 1–11. doi:10.1016/j.cub.2010.11.056. http://doi.org/10.1016/j.cub.2010.11.056

Dewar, A. D. M., Wystrach, A., Graham, P., and Philippides, A. (2015). Navigation-specific neural coding in the visual system of Drosophila. Bio Systems 136, 120–127. doi:10.1016/j.biosystems.2015.07.008. http://doi.org/10.1016/j.biosystems.2015.07.008

Hanesch, U., Fischbach, K. F., and Heisenberg, M. (1989). Neuronal architecture of the central complex in Drosophila melanogaster. Cell and Tissue Research 257, 343–366. doi:10.1007/BF00261838. http://doi.org/10.1007/BF00261838

Heinze, S. and Homberg, U. (2008). Neuroarchitecture of the central complex of the desert locust: Intrinsic and columnar neurons. The Journal of Comparative Neurology 511, 454–478. doi:10.1002/cne.21842. http://doi.org/10.1002/cne.21842

Ito, K., Shinomiya, K., Ito, M., Armstrong, J. D., Boyan, G., Hartenstein, V., et al. (2014). A Systematic Nomenclature for the Insect Brain. Neuron 81, 755–765. doi:10.1016/j.neuron.2013.12.017. http://doi.org/10.1016/j.neuron.2013.12.017

Lin, C.-Y., Chuang, C.-C., Hua, T.-E., Chen, C.-C., Dickson, B. J., Greenspan, R. J., et al. (2013). A Comprehensive Wiring Diagram of the Protocerebral Bridge for Visual Information Processing in the Drosophila Brain. Cell Reports 3, 1739–1753. doi:10.1016/j.celrep.2013.04.022. http://doi.org/10.1016/j.celrep.2013.04.022

Martín-Peña, A., Acebes, A., Rodríguez, J.-R., Chevalier, V., Casas-Tinto, S., Triphan, T., et al. (2014). Cell types and coincident synapses in the ellipsoid body of Drosophila. European Journal of Neuroscience 39, 1586–1601. doi:10.1111/ejn.12537. http://doi.org/10.1111/ejn.12537

Otsuna, H. and Ito, K. (2006). Systematic analysis of the visual projection neurons of Drosophila melanogaster. I. Lobula-specific pathways. The Journal of Comparative Neurology 497, 928–958. doi:10.1002/cne.21015. http://doi.org/10.1002/cne.21015

Otsuna, H., Shinomiya, K., and Ito, K. (2014). Parallel neural pathways in higher visual centers of the Drosophila brain that mediate wavelength-specific behavior. Frontiers in Neural Circuits 8. doi:10.3389/fncir.2014.00008. http://doi.org/10.3389/fncir.2014.00008

Pfeiffer, K. and Homberg, U. (2014). Organization and Functional Roles of the Central Complex in the Insect Brain. Annual Review of Entomology 59, 165–184. doi:10.1146/annurev-ento-011613-162031. http://doi.org/10.1146/annurev-ento-011613-162031

Phillips-Portillo, J. (2012). The Central Complex of the Flesh Fly, Neobellieria bullata: Recordings and Morphologies of Protocerebral Inputs and Small-Field Neurons. The Journal of Comparative Neurology 520, 3088–3104. doi:10.1002/cne.23134. http://doi.org/10.1002/cne.23134

Seelig, J. D. and Jayaraman, V. (2013). Feature detection and orientation tuning in the Drosophila central complex. Nature 503, 262–266. doi:10.1038/nature12601. http://doi.org/10.1038/nature12601

Wolff, T., Iyer, N. A., and Rubin, G. M. (2015). Neuroarchitecture and neuroanatomy of the Drosophila central complex: A GAL4-based dissection of protocerebral bridge neurons and circuits. The Journal of Comparative Neurology 523, 997–1037. doi:10.1002/cne.23773. http://doi.org/10.1002/cne.23705

Young, J. and Armstrong, J. (2010a). Building the central complex in Drosophila: The generation and development of distinct neural subsets. The Journal of Comparative Neurology 518, 1525–1541. doi:10.1002/cne.22285. http://doi.org/10.1002/cne.22285

Young, J. and Armstrong, J. (2010b). Structure of the adult central complex in Drosophila: organization of distinct neuronal subsets. The Journal of Comparative Neurology 518, 1500–1524. doi:10.1002/cne.22284. http://doi.org/10.1002/cne.22284
